# *Aphanomyces euteiches* Cell Wall Fractions Containing Novel Glucan-Chitosaccharides Induce Defense Genes and Nuclear Calcium Oscillations in the Plant Host *Medicago truncatula*

**DOI:** 10.1371/journal.pone.0075039

**Published:** 2013-09-23

**Authors:** Amaury Nars, Claude Lafitte, Mireille Chabaud, Sophie Drouillard, Hugo Mélida, Saïda Danoun, Tinaig Le Costaouëc, Thomas Rey, Julie Benedetti, Vincent Bulone, David George Barker, Jean-Jacques Bono, Bernard Dumas, Christophe Jacquet, Laurent Heux, Judith Fliegmann, Arnaud Bottin

**Affiliations:** 1 Laboratoire de Recherche en Sciences Végétales (UMR 5546), Université de Toulouse (UPS), Castanet-Tolosan, France; 2 UMR5546, CNRS, Castanet-Tolosan, France; 3 Laboratoire des Interactions Plantes-Microorganismes (UMR 441), INRA, Castanet-Tolosan, France; 4 Laboratoire des Interactions Plantes-Microorganismes (UMR 2594), CNRS, Castanet-Tolosan, France; 5 CERMAV, CNRS, Grenoble, France; 6 Division of Glycoscience, School of Biotechnology, Royal Institute of Technology, AlbaNova University Center, Stockholm, Sweden; Woosuk University, Republic of Korea

## Abstract

*N*-acetylglucosamine-based saccharides (chitosaccharides) are components of microbial cell walls and act as molecular signals during host-microbe interactions. In the legume plant *Medicago truncatula*, the perception of lipochitooligosaccharide signals produced by symbiotic rhizobia and arbuscular mycorrhizal fungi involves the Nod Factor Perception (NFP) lysin motif receptor-like protein and leads to the activation of the so-called common symbiotic pathway. In rice and *Arabidopsis*, lysin motif receptors are involved in the perception of chitooligosaccharides released by pathogenic fungi, resulting in the activation of plant immunity. Here we report the structural characterization of atypical chitosaccharides from the oomycete pathogen *Aphanomyces euteiches*, and their biological activity on the host *Medicago truncatula*. Using a combination of biochemical and biophysical approaches, we show that these chitosaccharides are linked to β-1,6-glucans, and contain a β-(1,3;1,4)-glucan backbone whose β-1,3-linked glucose units are substituted on their C-6 carbon by either glucose or *N*-acetylglucosamine residues. This is the first description of this type of structural motif in eukaryotic cell walls. Glucan-chitosaccharide fractions of *A. euteiches* induced the expression of defense marker genes in *Medicago truncatula* seedlings independently from the presence of a functional Nod Factor Perception protein. Furthermore, one of the glucan-chitosaccharide fractions elicited calcium oscillations in the nucleus of root cells. In contrast to the asymmetric oscillatory calcium spiking induced by symbiotic lipochitooligosaccharides, this response depends neither on the Nod Factor Perception protein nor on the common symbiotic pathway. These findings open new perspectives in oomycete cell wall biology and elicitor recognition and signaling in legumes.

## Introduction

The cell wall of pathogenic microorganisms is essential for cell integrity, morphogenesis, and stress resistance, and contains molecular structures which can be recognized as “non-self” by respective hosts, thereby triggering the activation of their immune systems [1], [2]. Obtaining insights into the composition, dynamics and recognition of microbial pathogen cell walls is thus of major importance for animal and human health as well as for plant protection. The Oomycete phylum comprises a high number of ecologically and economically important water molds. Many Oomycete species are pathogenic, such as the Saprolegniales *Saprolegnia parasitica*, which causes saprolegniosis in salmonids, and the Peronosporales *Phytophthora sojae* (*P. sojae*) which causes soybean root rot [3], [4]. Despite the fact that Oomycetes share some ecological and morphological traits with true fungi, they are in fact phylogenetically related to the Heterokont/Stramenopile Kingdom [5]. This evolutionary distance has led to molecular and cellular specificities, well-illustrated in the case of the *Phytophthora* genus where the presence of cellulose and the absence of chitin, *i.e.* the crystalline cell wall scaffold polymers, was demonstrated more than 60 years ago [6] and proposed as a discriminating taxonomic criterion [7]. Accordingly, it was recently demonstrated that oomycete-specific carboxylic acid amide fungicides target a cellulose synthase enzyme [8]–[10]. However, following the pioneering work on *Phytophthora* species, chitin was unequivocally detected via X-ray crystallography in some oomycete species [11]–[13]. Since then, the Oomycetes have been divided into two groups based on the nature of their crystalline scaffold polymers. The Leptomitales, which are early-diverging Oomycetes [5], contain both cellulose and chitin [11], whereas the late evolving Peronosporales seem to have retained cellulose only [7]. The Saprolegniales, which share common ancestry with the Leptomitales [5], have long been assumed to harbor a chitin/cellulose cell wall based on data obtained in *Achlya* and *Saprolegnia* [12], [13]. More recently, the expression of chitin synthase (CHS)-like genes was reported in diverse Oomycetes [10], [14]–[17]. Heterologous expression and *in vitro* biochemical assays have revealed that the *CHS2* gene from *Saprolegnia monoica* actually encodes a functional CHS enzyme. The latter is inhibited by nikkomycin Z, a structural analogue of the chitin synthase substrate UDP-GlcNAc, which further supports the occurrence of chitin in this microorganism [16].

*Aphanomyces euteiches* (*A. euteiches*) is a Saprolegniales responsible for important pea and alfalfa crop losses worldwide [18]. Recent studies suggest that, in contrast to *Saprolegnia* or *Achlya*, the dominating GlcNAc-based cell wall components in *Aphanomyces* species are structural non-crystalline chitosaccharides [14] comprising either 1,6-linked or 1,4-linked *N*-acetylglucosamine (GlcNAc) residues [19]. *A. euteiches* chitosaccharide biosynthesis was sensitive to nikkomycin Z, which caused hyphal growth arrest and bursting [14]. These findings demonstrated that chitosaccharides are involved in cell wall function and integrity in *A. euteiches*, and showed that GlcNAc-based polymers do not necessarily need to be crystalline in order to play a structural role in the cell walls of Oomycetes. This structural function might result from covalent association with cell wall glucans to form and stabilize a tridimensional network, as already shown in *Saccharomyces cerevisiae* and *Aspergillus fumigatus* (*A. fumigatus*) [20]–[22]. Although this hypothesis is supported by the observation that *A. euteiches* chitosaccharides can be solubilized by incubation of the cell wall with glucanases [14], experimental evidence for a covalent association is lacking.

During evolution, both animals and plants have developed the ability to recognize exogenous “non-self” compounds from aggressive microorganisms which trigger adapted immune responses in the hosts [1], [2]. In plants, the recognition of these compounds, also called elicitors, relies on specific receptors that bind to conserved molecular structures referred to as “Microbe/Pathogen-Associated Molecular Patterns” (MAMPs/PAMPs) [2]. Cell wall polymers of glucose (Glc) or GlcNAc, such as β-1,3;1,6-glucans or chitin, are sources of oligoglucosides or chitooligosaccharides (COs) respectively, which act as MAMPs in plants (reviewed in [2], [23], [24]). In the case of COs, the strongest inducers of plant defense exhibit a degree of polymerization (DP) of 6 to 8. Their perception depends on complexes of receptor-like proteins containing extracellular lysin motif (LysM) domains, which have been shown to mediate the binding of GlcNAc-containing ligands (reviewed in [25]–[27]). Subsequent transduction includes rapid cellular responses such as transmembrane ion fluxes, followed by the activation of defense-related gene expression programs [2], [24].

Interestingly, leguminous plants are able to establish mutualistic endosymbiotic associations with soil rhizobia, which depend on the recognition of specific bacterial lipochitooligosaccharides (LCOs) by host LysM receptor-like kinases (reviewed in [25], [28]). Cellular responses to rhizobial LCOs include a highly characteristic asymmetric oscillatory calcium signaling (known as spiking), and require the activation of a specific endosymbiotic signal transduction pathway, known as the common symbiotic pathway (CSP). This subsequently leads to the activation of symbiotic gene expression and a set of morphogenetic and organogenetic responses required for rhizobial intracellular colonization and nodule development. Recent findings suggest that LCOs may also be produced by arbuscular-mycorrhizal (AM) fungi and play a role in the establishment of their mutualistic symbiotic interaction with vascular plants [29]. In addition, COs with a DP of 4 or 5 present in AM spore exudates have recently been proposed to act as fungal symbiotic signals based on their ability to trigger AM-specific Ca^2+^ spiking dependent on the CSP in the model legume *Medicago truncatula* (*M. truncatula*) [30].

In *M. truncatula,* the LysM receptor-like kinase called NFP (for Nod Factor Perception) is required for perception of rhizobial LCO signals [31], whereas specific chitin receptor(s) have not yet been identified in this plant [32]. *M. truncatula* is a host for *A. euteiches*, with various ecotypes displaying different levels of susceptibility to this pathogen [33], [34]. We reported recently that a *M. truncatula nfp* mutant displays an increased susceptibility to *A. euteiches* [35]. This suggested that NFP is also involved in the perception of *A. euteiches* GlcNAc-containing PAMPs released from cell wall chitosaccharides. In order to examine this question, we are describing here the isolation and characterization of *A. euteiches* chitosaccharides and the evaluation of their biological activity in *M. truncatula* roots. We have monitored whole-tissue gene expression levels and nuclear calcium responses in the host root epidermis, and assessed the role of *NFP* or CSP genes in the host response to chitosaccharides.

## Results

### 1. The A. euteiches cell wall contains GlcNAc-based heteroglycans

#### 1.1. Association of the A. euteiches chitosaccharides with β-1,6-glucans

Our previous study [14] revealed that the *A. euteiches* chitosaccharides were most efficiently released from the cell wall by a bacterial enzymatic mixture enriched in β-1,6-glucanase activity (Westase™), suggesting that these compounds are closely associated with β-1,6-glucans. In order to gain insight into the underlying chemical linkages and the susceptibility of the chitosaccharides to enzymatic hydrolysis, cell wall samples were treated with several purified glycoside hydrolases with unique specificities. The enzymatic preparations were either commercial and, in certain cases, further purified, or purified directly from tobacco cell suspension cultures (see Materials and Methods). The extent of hydrolysis by chitinase and the amount of chitosaccharides released from the cell wall upon glucanase treatment were determined by comparison with the quantity of hexosamine liberated after acid hydrolysis of the initial cell wall sample, which was set at 100%. Only 6.4% of the chitosaccharides were hydrolyzed by endochitinase, indicating that a high proportion of these compounds are either inaccessible or resistant to this hydrolase, whereas exochitinase hydrolyzed approximately one third of the chitosaccharides (Table 1). When β-1,4- or β-1,3-endoglucanases were used, less than 10% of the chitosaccharides were released. However, incubation with β-1,6-endoglucanase strikingly released more than 96% of the chitosaccharides, thereby strongly suggesting the existence of covalent linkages between the chitosaccharides and β-1,6-glucans. Based on these observations, we propose to designate these heteroglycans as “glucan-chitosaccharides”.

**Table 1:** Percentage of chitosaccharides liberated by incubation of the cell wall with purified glycoside hydrolases^a^.

Endochitinase	6.4±1.4 A
Exochitinase	34.5±9.9 A
β-1,4-endoglucanase	8.3±0.7 A
β-1,3-endoglucanase	9.8±4.6 A
β-1,6-endoglucanase	96.5±0.5 B

a Data are ratios of liberated chitosaccharides to the total amount of chitosaccharides present in the starting cell wall material, and are mean percentages±S.E. of 3 independent experiments. Means for each treatment followed by a different letter are significantly different from each other (*P<*0.05). Enzymes were purified as described in Materials and Methods, except the β-1,4-endoglucanase from Megazyme which was used without further purification.

In response to microbial attack, plants secrete a variety of chitinases and glucanases that degrade the cell wall of the invading pathogen [36]. However, there are no reports to date that describe the production of β-1,6-glucanases by plant hosts. In order to investigate whether *A. euteiches* is able to secrete this type of enzymes, we measured various glucanase activities in culture filtrates of the pathogen. A low level of β-1,6-glucanase activity was detected in addition to β-1,3 and β-1,4 glucanase activities (Table S1). Analysis of the hydrolysis products by high-performance anion-exchange chromatography-pulsed amperometric detection (HPAEC-PAD) revealed the liberation of oligomers from pustulan, a linear β-1,6-glucan, thus indicating the presence of an endoactive β-1,6-glucanase (data not shown). In addition, two partial unigenes deduced from *A. euteiches* Expressed Sequence Tags [37], Ae_12AL6320 and Ae_1AL0240, were found to possess a glycoside hydrolase GH30 family domain (*E*-value<e^−30^) which occurs in most of the functionally-characterized β-1,6-glucanases according to the carbohydrate-active enzymes database (CAZY, [38]). Both unigenes were represented by at least one expressed sequence tag from an *A. euteiches* - *M. truncatula* interaction cDNA library, suggesting that the corresponding β-glucanase isozymes are produced *in planta*. Thus, *A. euteiches* itself might release glucan-chitosaccharides from its cell wall during the interaction with its host. Functional studies of the Ae_12AL6320 and Ae_1AL0240 gene products might reveal their possible involvement in this process.

#### 1.2. Isolation of glucan-chitosaccharide fractions from the cell wall

**Figure 1:**
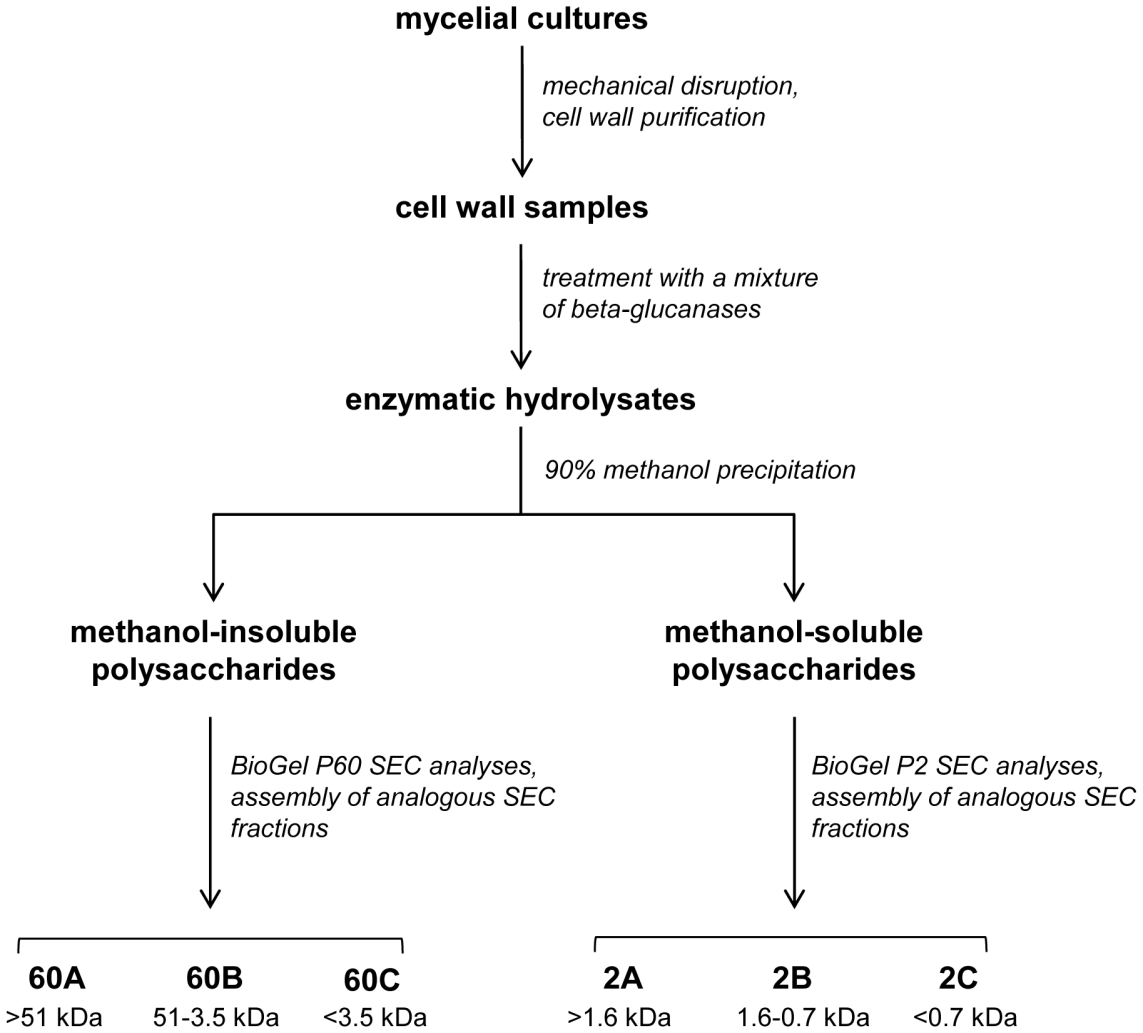
Preparation process of cell wall glucan-chitosaccharide fractions.

In order to optimize the release of chitosaccharides from the *A. euteiches* cell walls and to maximize the hydrolysis of glucan chains, cell wall samples were incubated with a mixture of three purified β-glucanases (Fig. 1 and Materials and Methods). After completion of the hydrolysis and precipitation of the enzymes by thermal inactivation, a sample of the hydrolysates was analyzed by ^15^N-cross-polarization/magic angle spinning nuclear magnetic resonance (NMR) to identify the hexosamines present in the solubilized chitosaccharides (Fig. S1). The only detected species was GlcNAc, which confirms that no glucosamine-based carbohydrates such as chitosan are present in the *A. euteiches* cell walls [14].

Cell wall samples were subjected to enzymatic hydrolysis and the resulting soluble carbohydrates were fractionated by methanol precipitation and separated by Size Exclusion Chromatography (SEC) on Biogel P60 and Biogel P2 matrices. This procedure led to the isolation of a total of six fractions, each corresponding to a different molecular weight (MW) range (Fig. 1). To evaluate their relative enrichment in chitosaccharides, the GlcNAc content of each fraction was compared to the GlcNAc content of the starting cell wall material, which was 12.2%. The SEC fractions 60C and 2C, which corresponded to low MW carbohydrates, were not analyzed further because they contained a high proportion of short-chain glucans and Glc generated by the action of glucanases, together with much lower amounts (8.5% and 5.5%, respectively) of GlcNAc. Carbohydrates in the remaining four fractions were analyzed after further purification by cation-exchange chromatography under acidic conditions to eliminate residual proteins (Table 2). Although they were significantly enriched in GlcNAc compared to the starting material, the major component in these fractions was Glc. This further supports our previous hypothesis that *A. euteiches* chitosaccharides are intimately associated with glucans in glucanase-resistant structures. In addition, it was not possible to further hydrolyze the carbohydrates from the various fractions using an additional enzymatic hydrolysis step or to separate the chitosaccharides from the glucans by affinity chromatography based on the specificity of the GlcNAc-binding lectin from *Datura stramonium* (data not shown). The glucan-chitosaccharides in the four fractions described in Table 2 were further analyzed to determine their chemical structure.

**Table 2:** Composition of glucan-chitosaccharide fractions obtained after size exclusion chromatography^a^.

Fraction	60A	60B	2A	2B
Apparent MW range (kDa)	>51	3.5–51	>1.6	0.7–1.6
Glc (%)	50.9	53.2	59.8	77.1
GlcNAc (%)	48.7	46.8	40.2	22.8
Proteins (%)	0.4	0.03	ND^b^	0.1

a Values are weight percentages of total measured material. Carbohydrates other than Glc and GlcNAc were not detected after HPAEC-PAD analysis of acid hydrolysates from the various fractions.

b ND: not detected.

#### 1.3. Characterization of a novel cell wall glucan-chitosaccharide structure

**Figure 2:**
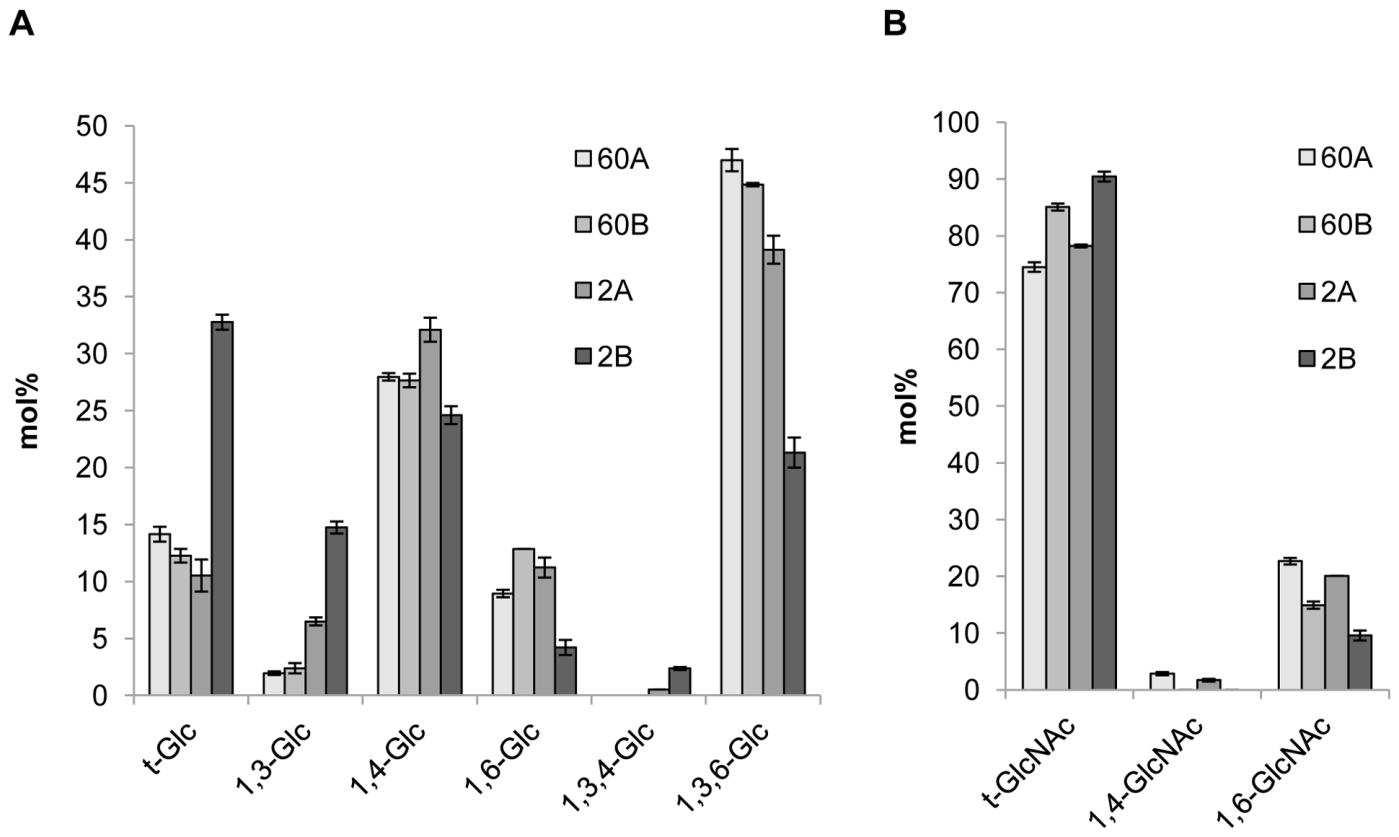
Linkage analysis of glucan-chitosaccharide fractions. The glycosidic linkages were identified by electron-impact mass spectrometry. Values are relative mole percentages (mol%) means ± S.D. of 4 technical replicates.

Linkage analysis of the glucan-chitosaccharide fractions revealed the presence of 1,3-, 1,4- and 1,6-linked glucosyl residues as well as of 1,3,4- and 1,3,6-linked glucosyl derivatives indicative of the presence of branching points along the glycan chains (Fig. 2A). Except for the 1,3,4-linked glucosyl residues, all derivatives have previously been identified in unfractionated alkali-insoluble cell wall fractions of *A. euteiches* [19]. Our data show that the 1,3,4-linked glucosyl residues occur in low amounts in fractions 2A and 2B only. Their detection is typically below background level when using total cell walls for linkage analysis [19] and becomes possible only after enrichment through specific fractionation steps, such as those used here. Fractions 60A, 60B and 2A showed similar profiles, with a high content of 1,3,6-linked glucosyl residues. This data indicates a high degree of branching, which is consistent with an increased resistance to glucanase hydrolysis. Fraction 2B, which corresponds to the lowest MW range, contained a higher proportion of terminal and 1,3-linked glucosyl residues compared to the other fractions, and a lower proportion of 1,3,6-linked glucosyl moieties, which indicates a less branched and shorter overall structure. Most of the GlcNAc derivatives detected were in the terminal position (Fig. 2B). The remaining non-terminal GlcNAc residues were essentially 1,6-linked while 1,4-linked GlcNAc residues were either minor or undetectable. The facts that the majority of GlcNAc residues are 1,4-linked in the *A. euteiches* cell wall [19] and that we used a glucanase mixture which was able to release all the chitosaccharides from the cell wall, indicate that most chitosaccharides containing 1,4-linked GlcNAc residues were lost during the purification of the fractions. We have recently shown that β-1,4-linked GlcNAc oligomers of DP above 8, which are poorly soluble in water, can occur in a metastable soluble state before irreversible precipitation [39]. We thus conclude that 1,4-linked GlcNAc in *A. euteiches* essentially occurs in poorly soluble carbohydrates which precipitated out during the purification, whereas the soluble glucan-chitosaccharides characterized here contain a high proportion of unusual 1,6-linked GlcNAc residues.

Fractions 2A and 2B were submitted to quadrupole time-of-flight (QTof) mass spectrometry analysis to demonstrate the existence of covalent linkages between Glc and GlcNAc residues. The presence of ammonium formate clusters in the lower MW fraction 2B, most likely originating from the SEC analysis, precluded the detection of any component of interest in this fraction, with the exception of a putative heterooligomeric GlcNAc-(Glc)_4_ species. In contrast, at least 16 Glc and/or GlcNAc oligomers, amongst which 14 putative hetero-oligomers containing both monosaccharides, were present in fraction 2A (Table 3). The covalent association of GlcNAc and Glc residues was unambiguously demonstrated by fragmentation analysis of 4 different selected species [GlcNAc-(Glc)_4_, (GlcNAc)_2_(Glc)_4_, (GlcNAc)_2_(Glc)_6_ and (GlcNAc)_3_(Glc)_6_], which allowed detection of diagnostic fragment ions corresponding to a GlcNAc-Glc heterodimer.

**Table 3:** Oligosaccharides identified in glucan-chitosaccharide fraction 2A by QTof-MS.

[M-H_2_O+H]^+^ ions^a^	[M+Na]^+^ ions^a^	Identity
204.09		GlcNAc
366.14		GlcNAc-Glc
407.17	447.17	(GlcNAc)_2_
528.19		GlcNAc-(Glc)_2_
	650.23	(GlcNAc)_3_
690.24		GlcNAc-(Glc)_3_
731.26	771.26	(GlcNAc)_2_(Glc)_2_
	812.29	(GlcNAc)_3_-Glc
852.29	892.29	GlcNAc-(Glc)_4_
	933.31	(GlcNAc)_2_(Glc)_3_
	974.34	(GlcNAc)_3_(Glc)_2_
1055.37	1095.37	(GlcNAc)_2_(Glc)_4_
	1136.39	(GlcNAc)_3_(Glc)_3_
	1177.40	(GlcNAc)_4_(Glc)_2_
	1257.42	(GlcNAc)_2_(Glc)_5_
	1419.47	(GlcNAc)_2_(Glc)_6_
	1622.55	(GlcNAc)_3_(Glc)_6_

a Data are *m/z* values.

**Figure 3:**
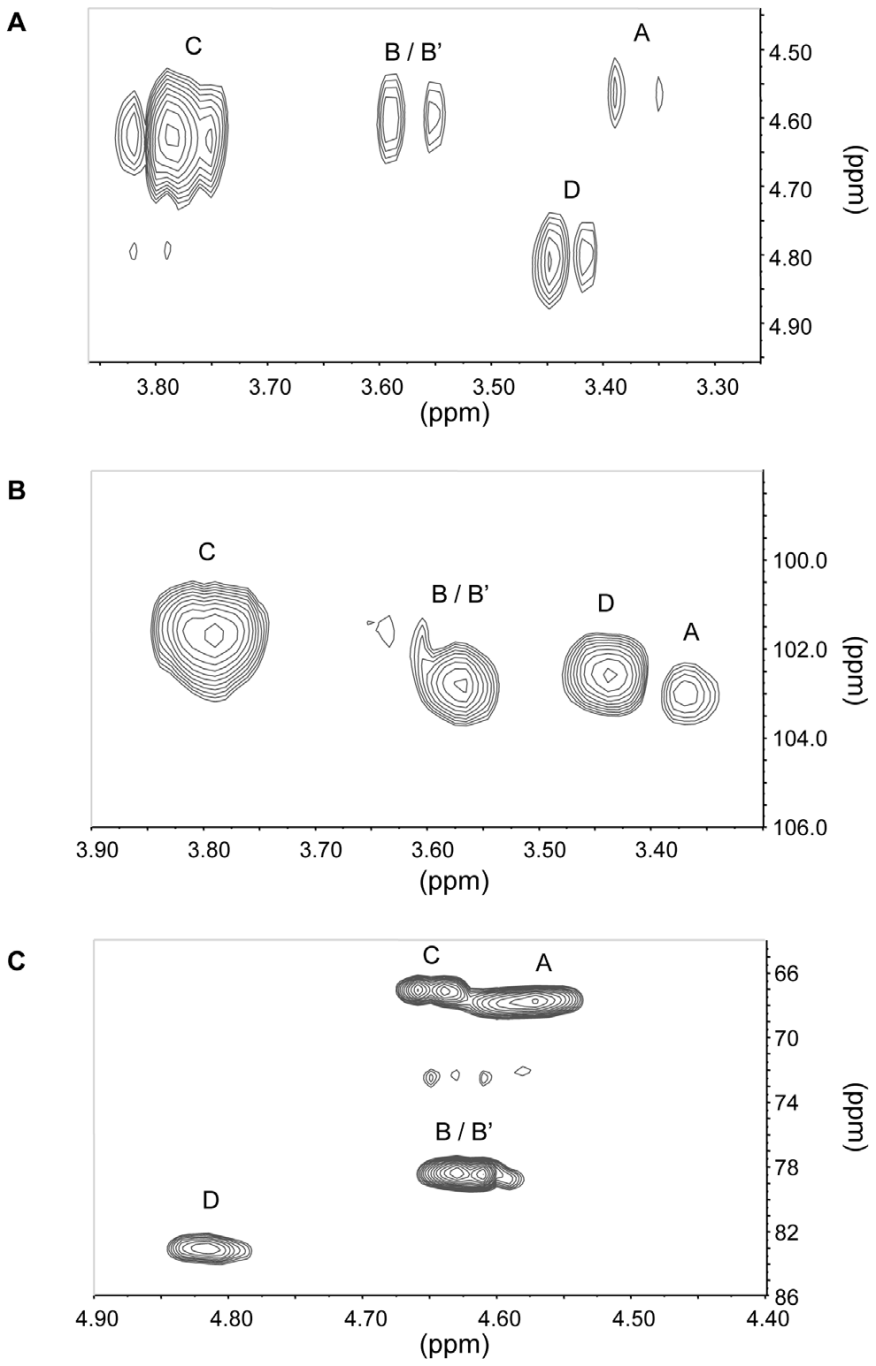
Two-dimensional liquid NMR spectra of glucan-chitosaccharide fraction 2B. **A**, Correlation spectroscopy (COSY) correlation (H-1/H-2) in the vicinity of anomeric protons; **B**, heteronuclear multiple bond coherence (HMBC) correlation in the vicinity of the C-1; **C**, heteronuclear multiple bond coherence (HMBC) correlation (C-3, C-4 and C-6/H-1) in the vicinity of the glycosidic linkages. Proton and carbon chemical shifts for units A, B/B’, C and D are given in Table 4.

**Figure 4:**
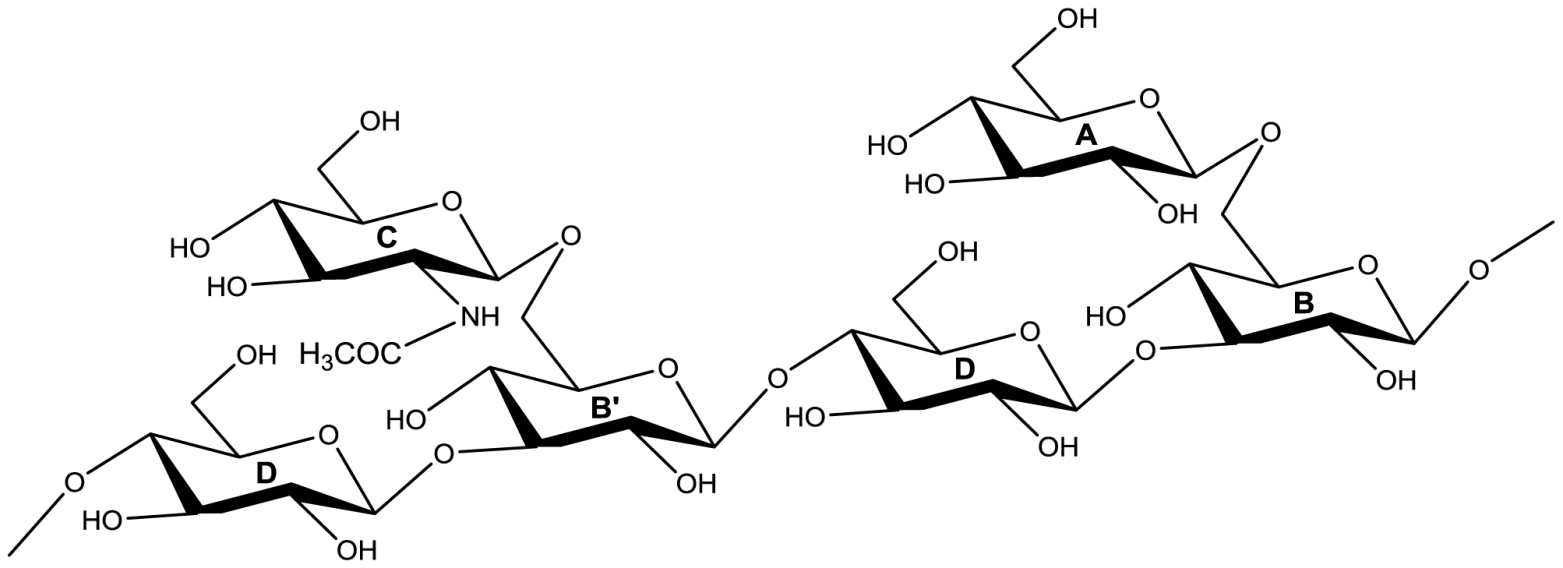
Model of core glucan-chitosaccharide molecular structure. The hexose residues are labeled with bold letters according to the legends of Fig. 3 and Table 4.

In order to determine the anomery of the glycosidic linkages and gain further insight into the molecular structures of the glucan-chitosaccharides, fractions 2A and 2B were subjected to two-dimensional (2D) liquid NMR analysis. The spectra obtained from the lower MW fraction 2B, which were less complex than those from fraction 2A, are presented on Fig. 3. Correlation spectroscopy (COSY) revealed 4 major units in the β configuration (A, B/B’, C and D correlations; Fig. 3A and Table 4). The identity and bonding of the Glc and GlcNAc residues within the glycan oligomer were resolved by the heteronuclear multiple bond coherence (HMBC) spectra (Fig. 3B and 3C). A glucan-chitosaccharide structure was deduced, further supporting the data obtained by permethylation linkage analysis (Fig. 4). The presence of alternating β-(1,3) and β-(1,4) linkages within the linear glucan chain was inferred from the absence of cross correlation between 1,3-linked glucosyl residues and between 1,4-linked glucosyl residues. The β-(1,3;1,4)-glucan chain is substituted by Glc or GlcNAc residues linked through 1→6 linkages to the β-(1,3)-linked glucosyl residues of the main chain (Fig. 4). 2D-NMR analysis of fraction 2A confirmed that this structure was also present in this fraction, most likely within larger glycans (data not shown). The nearly two-fold enrichment in GlcNAc of fraction 2A compared to fraction 2B (Table 2) suggests that the chitosaccharide substitutions on the main glucan chain are more frequent and larger in fraction 2A than in fraction 2B. This is supported by (i) the higher level of 1,3,6-linked glucosyl residues and the lower level of 1,3-linked glucosyl residues (Fig. 2A), indicative of a higher frequency of branching points on the 1,3-linked glucosyl residues of the main chain, and (ii) the higher level of 1,6-linked glucosyl and *N*-acetylglucosaminyl residues combined with a lower level of terminal Glc and GlcNAc (Fig. 2A and 2B), which supports the occurrence of longer branch chains with 1,6 linkages. When considering these data altogether, it can be concluded that the glucan-chitosaccharides from fractions 2A and 2B are fragments of larger branched glycans containing a linear β-(1,3;1,4)-glucan backbone where the β-(1,3)-glucosyl units are substituted by GlcNAc- or Glc-based chains through 1→6 branching linkages.

**Table 4:** Proton and carbon chemical shifts of glucan-chitosaccharide fraction 2B^a^.

	Unit				
	A	B	B’	C	D
H-1	4.57	4.61	4.61	4.64	4.82
C-1	102.7	101.47	101.47	101.27	102.5
H-2	3.37	3.57	3.57	3.78	3.42
C-2				55.57	
H-3		3.79	3.79		
C-3		84.23	84.23		
H-4					3.60
C-4					79.44
H-6		4.21, 3.64	4.27, 3.93		
C-6		68.89	68.04		

a Values are expressed in ppm. The hexose residues were labeled A to G in order of increasing chemical shift of their anomeric protons. B and B’ branching point units were differentiated on the basis of their H-6 and C-6 chemical shifts. Values of the free C-6 for units A, C and D are 60.65, 60.54 and 60.12 ppm.

### 2. *A. euteiches* glucan-chitosaccharide fractions induce defense genes and calcium oscillations in *M. truncatula*

#### 2.1. NFP-independent induction of defense-associated genes

The capacity of fractions 60A, 60B, 2A and 2B to activate the expression of defense-associated genes in wild type (WT) and *nfp* mutant seedlings of *M. truncatula* was investigated following a previously established experimental setup [39]. The root systems of hydroponically-grown seedlings were incubated for 4 h in the presence of each fraction (100 µg.ml^−1^). A chitin oligomer (CO) mixture with a mean DP of 6.8 [39] and the flagellin peptide elicitor (Flg22, [40]) were used as control elicitors.

**Figure 5:**
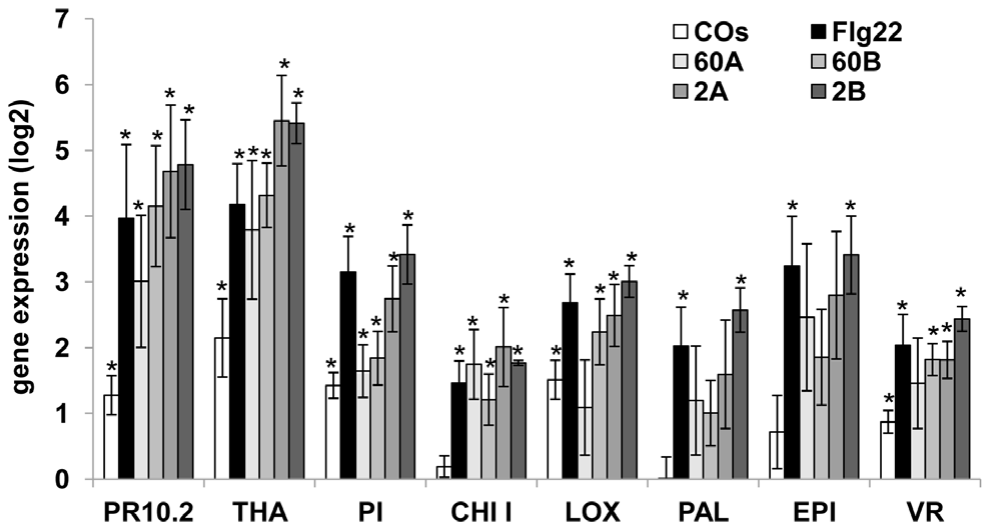
Expression of defense-associated genes in *Medicago truncatula* in response to glucan-chitosaccharide fractions. Gene expression in the seedling root system was analyzed by qRT-PCR after 4 h treatment with 100 µg.ml^−1^ fraction 60A, 60B, 2A or 2B. Chitooligosaccharides (COs) of mean degree of polymerization 6.8 [39] and Flg22 [40] were used as control elicitors at 20 µg.ml^−1^ and 1 µM, respectively. Defense-associated gene expression was standardized in each sample using three reference genes encoding an histone H3, a translation elongation factor 1-α and a ubiquitin family protein/phosphatidylinositol 3,4-kinase, as described in [39]. Mean values from three biological replicates (± S.E.) are given as log2 of fold-expression in elicited seedlings with respect to mock-treated seedlings. Asterisks above the bars indicate that gene induction in elicited seedlings was significant (*P*<0.05). PR10.2, pathogenesis-related protein 10; THA, thaumatin; PI, proteinase inhibitor; CHI I, class I chitinase; LOX, lipoxygenase; PAL, phenylalanine ammonia lyase; EPI, NAD dependent epimerase/dehydratase; VR, vestitone reductase.

The expression levels of pathogenesis-related genes (*PR10.2*, *THA*, *PI* and *CHI I*, encoding a pathogenesis-related protein 10, a thaumatin-like protein, a proteinase inhibitor, and a class I chitinase, respectively), or genes associated with oxylipin, phenylpropanoid, or flavonoid biosynthesis (*LOX*, *PAL*, *EPI* and *VR*, encoding a lipoxygenase, a phenylalanine ammonia lyase, an NAD-dependent epimerase/dehydratase, and a vestitone reductase, respectively) were measured by qRT-PCR in the treated roots. A significant induction (*P*<0.05) was observed for the PR10.2, THA, PI, LOX and VR markers in response to the CO mixture, while all genes analyzed responded to Flg22 (Fig. 5). All the glucan-chitosaccharide fractions significantly induced the four pathogenesis-related genes *PR10.2*, *THA*, *PI* and *CHI I.* In addition, fractions 60B, 2A and 2B induced the LOX and VR markers, and fraction 2B further induced the PAL and EPI markers, thereby inducing all the analyzed genes (*P*<0.05). This data show that all four fractions are active defense gene elicitors, with a tendency towards an increased number of responding genes the lower the MW of the fraction tested. Plants carrying allelic mutations in the *NFP* gene responded to the four fractions similarly to the WT line (Fig. S2). Thus, all four glucan-chitosaccharide fractions contain defense elicitors which are perceived by *M. truncatula* in an *NFP-*independent manner.

#### 2.2. NFP and CSP-independent induction of nuclear calcium signaling

Intracellular calcium signaling is central to many physiological responses in plants, including responses to symbiotic or pathogenic signals [2], [24], [25], [41]. Recently, it has been shown that germinating AM spore exudates contain short chain COs which are able to induce Ca^2+^ spiking responses in epidermal tissues of *M. truncatula* root organ cultures (ROCs) expressing a nuclear-targeted “cameleon” fluorescent Ca^2+^ reporter [30]. In this system, chitotetraose or chitopentaose are highly active at 10^−8^ M, whereas rhizobial LCOs are inactive at this concentration.

**Figure 6:**
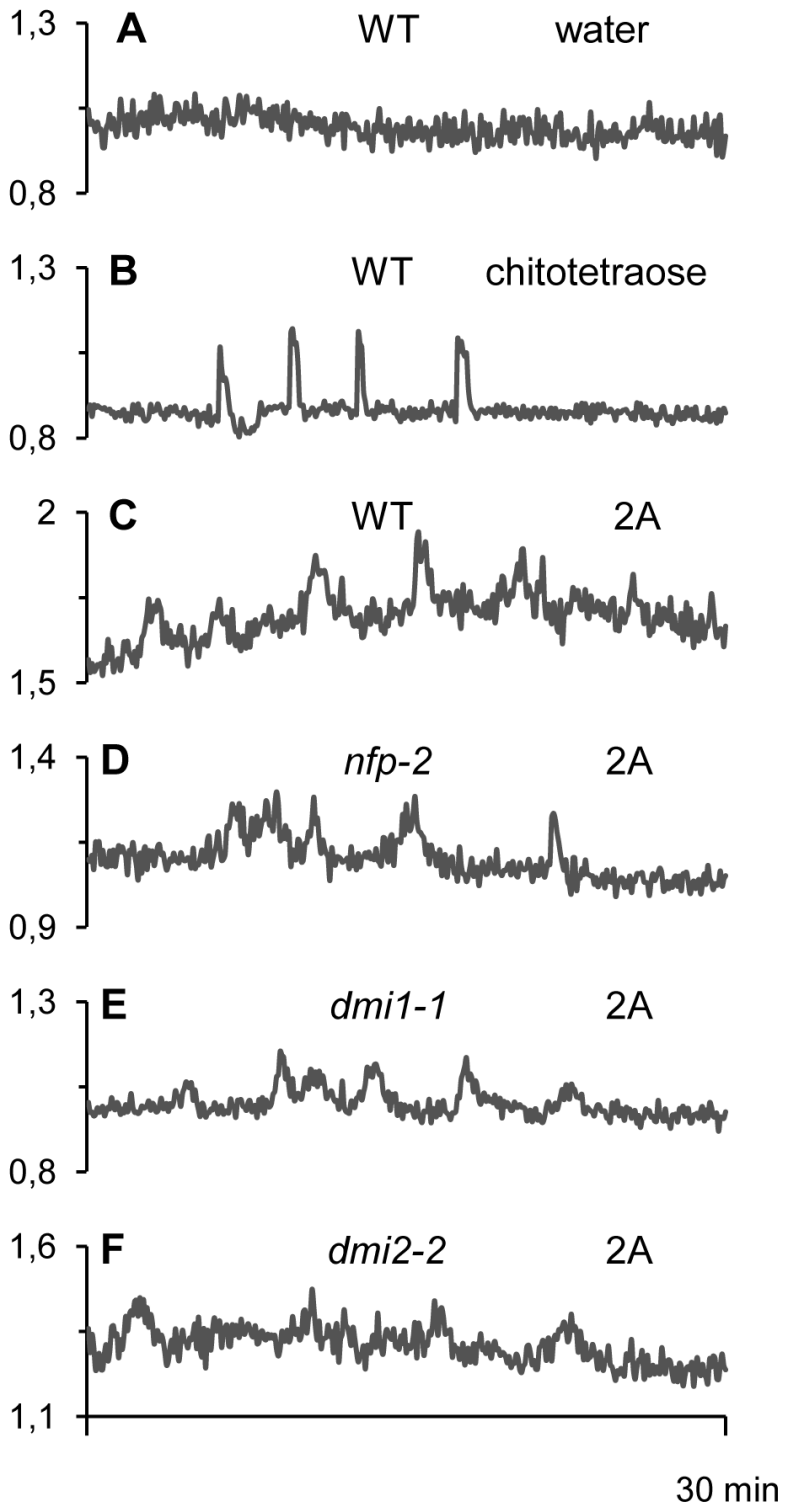
Nuclear Ca^2+^ variations in *Medicago truncatula* root epidermal cells in response to glucan-chitosaccharide fractions. Ca^2+^ variations were recorded in nuclei of epidermal cells of root organ cultures expressing the nuclear cameleon NupYC2.1 (see Materials and Methods) derived from either wild-type (WT) or mutant plants. **A**, WT root without treatment (negative water control). **B**, WT root treated with 10^−8 ^M chitotetraose. **C to F**, WT (**C**), or mutant (**D**, *nfp-2*; **E**, *dmi1-1*; **F**, *dmi2-2*) roots treated with 1 mg.ml^−1^ glucan-chitosaccharide fraction 2A. Roots were imaged every 5 s for 30 min, and data are presented as YFP-to-CFP ratios (arbitrary units).

In the experiment shown in Fig. 6B, 10^−8^ M chitotetraose, used as a positive control for Ca^2+^ signaling in the host root, induced a mean of 4 spikes during the 30 minutes following its application. Out of the four glucan-chitosaccharide fractions tested, only fraction 2A was able to trigger modifications of the nuclear Ca^2+^ concentration when applied at 1 mg.ml^−1^ (Table 5; Fig. 6C). Although these modulations are oscillatory, the individual peak profiles do not show the very rapid increase in nuclear Ca^2+^ concentration typical of the symbiotic signal-dependent spiking responses (compare Figs. 6B and 6C). In addition, there is high background noise in all the readouts and both peak frequency and peak width appear variable. Although fraction 2A was still able to elicit Ca^2+^ oscillations at 100 µg.ml^−1^, responses were less frequent at this lower concentration (data not shown). Thus concentrations of 1 mg.ml^−1^ were used routinely to ensure adequate Ca^2+^ responses. AM exudate- and chitotetraose-induced Ca^2+^ spiking are known to be independent of the rhizobial LCO receptor NFP, but do depend on the nuclear envelope-localized ion channel DMI1 and the plasma membrane receptor-like protein kinase DMI2, two essential components of the CSP [30], [42]. We took advantage of the availability of cameleon-expressing ROCs defective in *NFP*, *DMI1* or *DMI2* to examine to what extent these proteins were necessary for the Ca^2+^ oscillations observed in response to fraction 2A. The data show clearly that none of these 3 genes are required for 2A-elicited oscillations (Fig. 6D–F and Table 5). In conclusion, the Ca^2+^ oscillations observed in response to fraction 2A are clearly different from the symbiotic signal-dependent Ca^2+^ spiking responses, and require neither NFP (essential for the establishment of the rhizobial symbiosis in *M. truncatula)* nor the activation of the CSP (essential for the establishment of both rhizobial and arbuscular mycorrhizal symbioses in legumes).

**Table 5:** Induction of nuclear calcium oscillations in *Medicago truncatula* root epidermal cells by glucan-chitosaccharide fractions^a^.

cell wall fraction	plant genotype^b^	proportion of responsive root fragments	proportion of responsive cells
60A	WT	0/2	0/18 (0%)
60B	WT	0/2	0/21 (0%)
2A	WT	3/3	25/31 (80%)
2B	WT	0/3	0/29 (0%)
2A	*nfp-2*	2/2	11/17 (65%)
2A	*dmi1-1*	2/2	20/23 (87%)
2A	*dmi2-2*	2/2	15/20 (75%)

a Two to three root fragments from cameleon-expressing root organ cultures where treated by glucan-chitosaccharides at a concentration of 1 mg.ml^−1^. The ratio of YFP to CFP light emissions from eight to eleven cells per root fragment was monitored over the time. Cells were counted responsive if at least 2 oscillations were observed over the 20–30 min imaging period. A root fragment was considered to be responsive when there was at least one responsive cell.

b All genotypes are in the Jemalong cultivar, A17 accession.

## Discussion

The structural cell wall polymers of some oomycetes were described several decades ago as corresponding essentially to cellulose, β-(1,3;1,6)-glucans [7], and minute amounts of chitin in certain species [11]–[13]. However, knowledge of the oomycete three-dimensional cell wall network and the covalent associations between the various polymers is still lacking. With the use of β-glucanases and mass spectrometry analyses, we show here that *A. euteiches* chitosaccharides are covalently associated to β-1,6-glucans. Whereas Glc-GlcNAc heteroglycans have only been shown to occur in the true fungi *Saccharomyces cerevisiae* and *A. fumigatus* [20]–[22], we provide here the first evidence of their existence in an Oomycete species. In addition, our data reveal for the first time the occurrence of a novel branched heteroglycan structure that consists of a glucan backbone with alternating β-1,3 and β-1,4 linkages.

β-(1,3;1,4)-glucans are major cell wall polysaccharides of the *Poaceae* plants and occur in lichen fungal symbionts where they are designated as lichenan or lichenin, as well as in the fungal pathogens *A. fumigatus* and *Rhynchosporium secalis* (reviewed in [43]). With only a few exceptions, such as *A. fumigatus* in which they consist of alternating 1,3- and 1,4-linked glucosyl residues [21], these non-crystalline polysaccharides are typically composed of cellotriosyl or cellotetraosyl units linked by β-1,3 glucosidic bonds. It has been proposed that they might tether fibrils of the cell wall scaffold polymers in plants [43]. In *A. fumigatus*, the β-(1,3;1,4)-glucan can be released from the alkali-insoluble fraction of the cell wall by β-(1,3)-glucanases, suggesting that it is covalently bound to β-(1,3)-glucan, a major structural polysaccharide in this organism [21]. Thus, β-(1,3;1,4)-glucan appears to have a structural role in plants and fungi, and now also in Oomycetes. However, the glycan structure that we have identified in *A. euteiches* presents a major difference with respect to the plant and fungal β-(1,3;1,4)-glucans characterized to date, since it is highly branched and contains GlcNAc residues, as opposed to its plant and fungal counterparts, which are typically linear homopolymers.

Although the *A. euteiches* chitosaccharides are essential for cell wall integrity, their structure is not crystalline [14]. Our data show that a fraction of the *A. euteiches* cell wall chitosaccharides is part of a highly branched heteroglycan composed of Glc and GlcNAc. The chitosaccharides within this complex polysaccharide may have a protective role against plant defense glucanases. This hypothesis is in line with the observation that the large, glucanase-resistant glycans in fractions 60A, 60B and 2A, contain the highest proportion of GlcNAc residues (Table 2). Furthermore, the presence of 1,6-linked *N-*acetylglucosaminyl residues would render part of the chitosaccharides resistant to plant chitinases, an assumption confirmed by the inability of an endochitinase to efficiently release chitosaccharides from the cell wall (Table 1). Thus, it can be proposed that chitosaccharide-containing domains of the *A. euteiches* cell wall are generally not prone to hydrolysis by plant defense glycanases. The complex, enzyme-resistant architecture of these domains is likely to confer a selective advantage to this pathogenic microorganism.

The wealth of data which has now been accumulated on the *A. euteiches* cell wall [14], [19], including the present study, allows us to revisit earlier assumptions concerning oomycete cell walls. It has sometimes been inferred that the Peronosporales *Lagenidium*, *Phytophthora*, *Plasmopara* or *Pythium* contain chitin, either because cell surface labeling was observed when using lectin conjugates of either chitinase or GlcNAc-specific wheat germ agglutinin, or because GlcNAc was released after incubation of cell wall samples with chitinase [15], [44]–[48]. Since these reports did not further substantiate the occurrence of crystalline GlcNAc-based polysaccharides via X-ray analysis, it is possible that the detected compounds corresponded to chitosaccharides similar to those we are describing in *A. euteiches*. Eventually, these compounds may become detectable only after specific enrichment, as shown here for the 1,3,4-glucosyl moieties identified in some *A. euteiches* glucan-chitosaccharide fractions. Indeed, 1,3,4-linked glucosyl and GlcNAc residues were not detected in *Aphanomyces* and *Phytophthora* species, respectively, in a recent, more global cell wall analysis of Oomycetes [19]. The involvement of very minor chitosaccharide components in cell wall function would reconcile the inability to detect chitin in many Oomycetes [7] despite the presence and expression of *CHS*-like genes [10], [14], [15] and the sensitivity of certain Oomycetes to CHS inhibitors ([49], and A. Bottin, unpublished results). A striking example of a very minor cell wall compound having an essential role in cell wall integrity is the case of *Saprolegnia monoica* chitin, which represents only 0.7% of the cell wall polysaccharides [12], [16]. Alternatively, some oomycete *CHS*-like genes might have evolved essential functions other than the biosynthesis of structural GlcNAc-based polymers, such as the biosynthesis of glycoprotein glycans. Because of the high impact of oomycete diseases on crop and aquatic animal production, and the limits of targeting only cellulose biosynthesis to combat these pests [9], [10], it is now important to study the structure-activity relationship of oomycete CHS-like enzymes, which form a distinct group in CHS phylograms [50], [51]. This should shed new light on their putative function as cell wall biosynthetic enzymes, and may allow the identification of new key targets for herbicides and fungicides with a narrower spectrum and reduced ecotoxicity.

Because cell wall polymers are sources of highly active PAMP molecules and chitosaccharides can play major roles in the molecular dialogues between plants and microorganisms [25]–[27], we decided to examine the elicitor activity of glucan-chitosaccharide fractions in *M. truncatula* by using a previously established experimental setup [39]. All the fractions induced the expression of a variety of defense-associated marker genes at a concentration of 100 µg.ml^−1^, which is well in the range of concentrations typically used for assaying elicitor activity of glycan mixtures [52]–[54]. The highest MW range fraction 60A appeared the least active and the lowest MW range fraction 2B the most active of the four fractions tested. However, this difference was not pronounced and might simply reflect steric hindrance affecting diffusion of large glucan-chitosaccharide molecules through the host plant cell wall, rather than specific distribution of elicitor molecules amongst the various fractions. Oomycete or fungal cell wall fragments with elicitor activity are typically homo-oligomers with a minimal DP of 5 (*e.g.* branched *Alternaria alternata* or *P. sojae* oligoglucan elicitors) or 6 (*e.g.* chitin fragments) [23]. No such homo-oligomeric molecules were detected here using Qtof MS. This, combined with the very low levels of 4-linked GlcNAc residues in the various fractions, as evidenced by our linkage analyses, suggests that the elicitor activity of the glucan-chitosaccharide fractions is not due to the presence of chitin oligomers (COs). Thus, it can be inferred that the biological activity of the *A. euteiches* glucan-chitosaccharides is associated with the Glc-GlcNAc heterooligomers detected by QTof MS in fraction 2A. A definitive proof of the activity of these compounds would require chemical synthesis of candidate molecules. However, chemical synthesis of such complex structures as those described here represents a major challenge.

It has been shown that the release of glucan PAMPs by oomycete pathogens is mediated, at least in part, by the action of plant defense endoglucanases [55]. The most active PAMP in soybean is a heptaglucan from *P. sojae* consisting of 5 β-1,6-linked glucosyl residues, two of which being substituted by glucosyl units at their C-3 [56]. This molecule most likely corresponds to a plant glucanase-resistant structure [57], not only because it is branched, but also because plants are not known to produce β-1,6-glucanase. Here, we show that only β-1,6-glucanase treatment efficiently releases chitosaccharides from the *A. euteiches* cell wall. Thus, in the natural interaction between *A. euteiches* and its host plant, chitosaccharides may only be liberated by β-1,6-glucanases produced by the microorganism itself. This would not be an unprecedented phenomenon, since it has already been shown that *P. sojae* releases glucan elicitors when it germinates from zoospores [58]. While these *P. sojae* elicitors are predicted to arise from the action of β-1,3-endoglucanases, we reveal here the existence of a β-1,6-endoglucanase activity secreted by *A. euteiches* during saprophytic growth, which might be involved in the release of chitosaccharide elicitors from the cell wall.

The use of cameleon-expressing roots as reporters for monitoring changes in calcium concentrations revealed for the first time in legumes that a cell wall fraction from a microbial pathogen is able to generate an oscillatory response within the nucleus of root epidermal cells. To date, nuclear oscillatory Ca^2+^ signaling has only been described in legumes in response to symbiotic chitinaceous signals such as LCOs and short-chain COs [25], [30], [59]. To our knowledge, only one other study has reported calcium oscillatory responses to a pathogenic elicitor, namely nuclear oscillations in leaf cells of the non-legume plant *Nicotiana benthamiana* treated with cryptogein, an oomycete proteinaceous elicitor of cell death and defense [60]. In general, calcium signaling in response to pathogenic elicitors has been described as a single cytoplasmic Ca^2+^ transient of variable profile, duration and intensity [41], [61], [62]. Indeed, a single transient was also observed in soybean and *Nicotiana sp.* suspension-cultured cells expressing the bioluminescent Ca^2+^ reporter aequorin following treatment with AM spore exudates containing symbiotic-specific signals [63], [64]. These apparent discrepancies are most likely the consequence of the different methods used for monitoring changes in the levels of intracellular calcium. Only the use of cameleon-type reporters targeted to specific compartments such as the cell nucleus allows the monitoring of individual cell responses using FRET-based confocal microscopy. In contrast, the use of aequorin-based reporters in cell suspension cultures leads to an averaging of the bioluminescent signal from the entire cell population, and is therefore unable to detect oscillatory responses in individual cells.

Interestingly, close examination of the *Aphanomyces* cell wall-elicited oscillations reveals that they do not have the asymmetric profile characteristic of the Ca^2+^ spikes induced by CO/LCO signals. Furthermore, the use of mutants (*dmi1* and *dmi2*) defective in the common symbiotic signaling pathway has shown that an alternative pathway must be involved in activating this novel oscillatory response. Altogether, these data strongly suggest that the mechanism regulating Ca^2+^ release into the nuclear compartment in response to the cell wall fraction of a pathogen is different from that triggered by symbiotic signals. In addition, it must be underlined that Ca^2+^ oscillations in *M. truncatula* roots were observed only in response to *A. euteiches* glucan-chitosaccharide fraction 2A. Since all four fractions were able to induce defense gene expression, it is not possible to directly correlate defense responses with calcium signaling. Therefore the full biological significance of this Ca^2+^ response will require the identification of the specific chemical elicitor, presumably present at higher levels in fraction 2A, as well as the analysis of mutant lines defective in this particular mode of Ca^2+^ signaling.

In conclusion, we provide a significant advance in eukaryotic cell wall biology by describing a novel oomycete structural component, a branched glucan-chitosaccharide from *A. euteiches*. In addition, we unequivocally show that cell wall glucan-chitosaccharide fractions are defense gene elicitors, and that they can trigger nuclear-associated calcium oscillations independent of the well-characterized CSP signal transduction pathway used by endosymbiotic microbes. The fact that the activation of defense genes by glucan-chitosaccharide fractions was independent of NFP suggests that they are perceived by one or several other receptors, which might belong either to the LysM or glucan-binding protein families [32], [65], for which most members have unknown biochemical functions in *M. truncatula*. Furthermore, the loss of resistance in *nfp* mutants suggests a regulatory role for *NFP* in immunity, rather than direct elicitor recognition. Taken together, our findings pave the way for a better understanding of the discrimination between beneficial and detrimental microorganisms by legumes.

## Materials and Methods

### 1. Biological material

*Aphanomyces euteiches* (Drechsler) strain ATCC201684 was maintained and induced to sporulate as described in [14]. It was grown in synthetic medium for the production of mycelium, or in glucose-yeast extract medium for the measurement of secreted enzyme activities, as described in [39] and [14], respectively. The mycelium was harvested by filtration on a sintered glass filter, washed with cold distilled water, frozen and stored at −80°C in 10-gram wet weight aliquots. BY2 tobacco cell suspension cultures and *M. truncatula* (Gaertn.) Jemalong A17 WT, *nfp-1* and *nfp-2* allelic mutant seedlings [31], [66], were grown as described in [67] and [39] respectively. Transgenic ROCs from Jemalong A17 (WT and derived mutants *nfp-2, dmi1-1, dmi2-2*) expressing the 35S:NupYC2.1 construct were obtained previously [42] and grown according to [30].

### 2. Reagents and enzymes

All reagents were of analytical grade. Enzymatic and chemical hydrolyses, and chromatographic analyses, were performed using Ultra-High Quality (UHQ) water as a solvent. Flg22 peptide, *Bacillus amyloliquefaciens* cellulase, and Westase™, were purchased from Proteogenix (France), Megazyme International (Ireland), and Takara Bio (Japan) respectively. Glycoside hydrolase activities were determined by incubation of enzyme samples at 40°C during 30 minutes (glucanase activity) or 2 h (chitinase activity) in 100 mM sodium acetate pH 5, 0.5 M NaCl, 5 mM phenylmethanesulfonylfluoride, 0.1% (v/v) Triton X100, in the presence of 0.5% (w/v) carboxymethylcellulose (Merck) for β-1,4-glucanase measurement, 0.5% (w/v) *Alcaligenes faecalis* curdlan (Sigma-Aldrich) for β-1,3-glucanase measurement, 0.5% pustulan (Calbiochem) for β-1,6-glucanase measurement, and 1% colloidal chitin prepared according to [68] for chitinase activity. The free sugar reducing ends were quantified by colorimetry [69], and the endo- or exo-action of the enzymes was assessed by HPAEC-PAD analysis of the hydrolysates as described in [14]. Specific activities were calculated after measuring protein contents according to [70], and expressed either in nkat.mg^−1^ or in U.mg^−1^, where 1 U is the amount of enzyme required to release 1 µmol of glucose reducing-sugar equivalents per min at 40°C. Purity of the enzymes was checked by measuring the various glycoside hydrolase activities using the assays described above. The data showed that the cellulase from Megazyme was a pure β-1,4-endoglucanase while Westase contained both β-1,6-endoglucanase and β-1,3-exoglucanase activities, which were purified as follows: 1 gram of powder was suspended in 100 ml of 10 mM Tris-HCl pH 8, incubated 1 h at room temperature under constant stirring, and centrifuged at 3,500 *g* for 10 minutes. The supernatant was loaded on a 25 ml diethylaminoethyl-Sepharose column (Biorad) equilibrated in 10 mM Tris-HCl pH 8 at room temperature at 40 ml.h^−1^, and both activities were eluted with 100 ml of a 0 to 1 M NaCl gradient. The eluate was dialyzed for 4 h at 4°C against 1 L of 10 mM Tris-HCl pH 8 containing 1 M ammonium sulphate in dialysis tubes with a 1 kDa molecular weight cut-off (MWCO). The dialysate was then analyzed on a 10 ml phenylsepharose column (Biorad) equilibrated in 10 mM Tris-HCl pH 8 containing 2 M ammonium sulphate, at 40 ml.h^−1^ and at room temperature. After a wash with 30 ml of the same buffer, the β-1,3-exoglucanase activity was eluted with 30 ml water, then the β-1,6-endoglucanase activity was eluted with 30 ml of 6 M urea. Urea was eliminated by dialysis overnight at 4°C against 1 L water in dialysis tubes with a 1-kDa MWCO.

Endo-β-1,3-glucanase, endochitinase and exochitinase were purified from 12-day old BY2 tobacco suspension-cultured cells grown as described in [67]. Twenty grams fresh cell weight were ground in liquid nitrogen with a pestle in a mortar, the powder was resuspended in 150 ml of 100 mM sodium acetate pH 5, 0.5 M NaCl, 5 mM phenylmethanesulfonylfluoride, 0.1% (v/v) Triton X100, and incubated 30 minutes at 6°C under constant stirring. The sample was then centrifuged at 2,500 *g* for 15 minutes at 4°C, and the pellet was extracted a second time following the same procedure, resulting in 30 ml of pooled supernatants. Ammonium sulfate was added up to a 20% (w/v) concentration, the extract was incubated for 2 h at 0°C, then centrifuged at 10,000 *g* for 30 minutes at 4°C. The supernatant was dialyzed overnight at 4°C against 30 L water in dialysis tubes with a 10–12 kDa (MWCO). The dialysate was supplemented with sodium acetate pH 5 (20 mM final concentration), then centrifuged at 10,000 *g* for 30 minutes at 4°C. The supernatant was analyzed on a 25 ml carboxymethyl-Sepharose column (Biorad) equilibrated in 20 mM sodium acetate pH 5 at 40 ml.h^−1^ at room temperature. Exochitinase activity was not retained on the column and recovered in the flow-through. Endochitinase and β-1,3-endoglucanase activities were recovered using 100 ml of a 0 to 2 M NaCl gradient, the former being eluted at 0.25 M NaCl and the latter at 1.3 M NaCl. All enzyme samples were stored at −20°C.

### 3. Preparation and enzymatic analysis of A. euteiches cell walls

The 50 ml hardened steel grinding bowl of a vibratory disc mill RS200 (Retsch) was pre-cooled in liquid nitrogen, then 10-gram aliquots of frozen mycelium were added to the bowl and ground each 3 times for 15 sec at 1,500 rpm. The powder resulting from 150 grams of mycelium was resuspended in 100 ml of 200 mM potassium phosphate pH 6.8. After 20 minutes incubation at room temperature under constant stirring, the sample was centrifuged at 1,000 *g* for 10 minutes at 15°C. All further centrifugation steps were performed in the same conditions. The pellet was resuspended in the same volume of phosphate buffer, further incubated for 20 minutes and centrifuged again. This procedure was repeated twice using phosphate buffer, 3 times using deionized water, twice using methanol/chloroform (2 v/1 v), and then 6 times using deionized water. It was further purified by extraction with a phenol-acetic acid-water (PAW) mixture (2∶1∶1, w/v/v) as described in [71]: the pellet was resuspended in 87.5 ml of PAW solution and incubated 16 h at room temperature under constant stirring before centrifugation. The pellet was resuspended in the same volume of deionized water, further incubated for 20 minutes and centrifuged again. This procedure was repeated 5 times using deionized water, and twice using 80% ethanol, and the samples were stored in 80% ethanol at −80°C. The Glc, hexosamine and protein contents were determined as described in [14]. For enzymatic analysis using the pure enzymatic preparations previously described (section 4.2.), cell wall aliquots (20 mg) were incubated in 20 ml of 100 mM sodium acetate pH 5.2 at 40°C for 20 h under constant stirring in the presence of either 4 U endochitinase, 6 U exochitinase, 12 U β-1,4-endoglucanase, 10 U β-1,3-endoglucanase or 10 U β-1,6-endoglucanase, respectively. Hydrolysis was stopped by incubation at 100°C for 10 minutes and the cell wall residue was collected by centrifugation at 3,500 *g* for 10 minutes at 4°C. The hexosamine content of the supernatant was measured by HPAEC-PAD analysis after acid hydrolysis as described in [14], and the percentage of chitosaccharides liberated from the starting cell wall sample was then calculated. Statistical significance of the differences observed between the various enzyme treatments for 3 independent experiments was determined by a Student *t*-test.

### 4. Preparation of glucan-chitosaccharide fractions

Three cell wall samples prepared as described above were further treated with 1 M NaOH to remove residual proteins, following the same procedure as for PAW extraction. The obtained deproteinized cell wall samples were composed exclusively of Glc and GlcNAc, the latter accounting for 12.2±0.75 (S.E.) % of dry weight. Cell wall aliquots (100 mg) were incubated in 40 ml of 100 mM sodium acetate pH 5.2 at 40°C for 20 h under constant stirring in presence of a mixture of the enzymatic preparations previously described (section 4.2.), containing 10 U β-1,3-exoglucanase, 60 U β-1,6-endoglucanase, 60 U β-1,3-endoglucanase and 80 U β-1,4-endoglucanase. After centrifugation at 3,500 *g* for 20 minutes at 4°C, the supernatants were incubated at 100°C for 10 minutes to inactivate and precipitate the enzymes, and centrifuged again under the same condition. The supernatants were concentrated ten-fold in a Büchi rotavapor at 30°C, then methanol was added up to a concentration of 90% (v/v). The samples were incubated overnight at −20°C and centrifuged at 3,500 *g* for 20 minutes at 4°C, resulting in 90% methanol-soluble (supernatants) and -insoluble (pellets) fractions. The supernatants were concentrated in a Büchi rotavapor at 30°C before being diluted to 1 ml in 100 mM ammonium formate pH 6.8, whereas the pellets were directly dissolved in 4 ml of the same buffer. The samples were analyzed separately by size exclusion chromatography (SEC) in 100 mM ammonium formate pH 6.8 on a Bio-Gel P-60 (90% methanol-insoluble fractions) or a Bio-Gel P-2 (90% methanol-soluble fractions) fine column (Biorad), respectively. The columns were calibrated with dextran standards and run at room temperature. Gel volumes, column sizes and flows were 120 ml, 2.6×23 cm and 40 ml.h^−1^ for Bio-Gel P-60 SEC, and 100 ml, 2.6×19 cm and 50 ml.h^−1^ for Bio-Gel P-2 SEC, respectively. Chromatographic fractions from different SEC analyses were harvested and pooled, resulting in fractions 60A, 60B and 60C obtained from Bio-Gel P-60 SEC analyses and corresponding to molecular masses of more than 51 kDa, from 3.5 to 51 kDa, and less than 3.5 kDa, respectively, and in fractions 2A, 2B and 2C obtained from Bio-Gel P-2 SEC analyses and corresponding to molecular masses of more than 1.6 kDa, from 0.7 to 1.6 kDa, and less than 0.7 kDa, respectively. The fractions were freeze-dried 2 times in order to concentrate them and eliminate ammonium formate. The Glc and hexosamine contents were determined by HPAEC-PAD analysis after acid hydrolysis as described in [14], and proteins were measured by the improved Bradford dye-binding procedure using the recommendations and reagents of the protein assay kit from Biorad. Residual proteins in fractions 60A, 60B, 2A and 2B were removed by anion exchange chromatography on a 10-ml AG50 Dowex H^+^ column equilibrated in 10 mM HCl at 20 ml.h^−1^ at room temperature.

### 5. Characterization of the glucan-chitosaccharide fractions

Monosaccharide linkage analysis was performed by methylation with methyl iodine in sodium hydroxide and dimethyl sulfoxide as described in [72]. Partially methylated alditol acetates were analysed by GC coupled to electron-impact MS as described in [19]. Mass spectrometry analysis of glucan-chitosaccharide fractions was performed by accurate mass measurement, using a Xevo-G2 QTof mass spectrometer (Waters) operated in Electron Spray Ionization (ESI) positive mode. The capillary voltage was set at 3.0 kV, the cone at 30 V, and collision energy at 5 eV or 20 eV for MS or MS/MS experiments respectively. The source and desolvation temperatures were 110°C and 300°C respectively, and the desolvation and nebulizer flows were 650 L/h and 20 L/h respectively. Leucine-Enkephalin was used for the lock mass of the lock spray, and ions were scored in the range from 150 to 1,800 Da. Solid-state NMR analysis was performed according to [73]. Liquid NMR spectra were recovered in D_2_O at 298°K on a Bruker Avance 400. The ^1^H and ^13^C-NMR assignments were based on homonuclear ^1^H-^1^H and heteronuclear ^1^H-^13^C correlation experiments (correlation spectroscopy COSY, heteronuclear multiple bond coherence HMBC, heteronuclear multiple quantum coherence HMQC).

### 6. Bioassays

Measurement of the expression of defense-associated genes in roots of *M. truncatula* whole seedlings was done according to [39]. The significance of gene induction by the various elicitor fractions (Fig. 5), and of differential gene regulation in the *nfp-1* or *nfp-2* mutant backgrounds (Fig. S2), was determined for 3 biological replications by Student *t*-tests. Monitoring of calcium oscillations in epidermal cells from ROCs was performed as described in [30]: young 3^rd^-order lateral roots grown for two weeks under Biofolie membrane (to limit root hair development) were excised, placed in a microchamber and treated with 100 µl of the solution to be tested. Confocal FRET-based ratio imaging for detecting and plotting relative changes of nuclear Ca^2+^ levels corresponding to YFP-to-CFP fluorescence intensity changes over time [74] was performed according to [59] using a Leica TCS SP2 AOBS confocal laser-scanning microscope equipped with a HCX PL APO 40X 0.85 dry objective. Fluorescence intensity for both the CFP and YFP moieties of NupYC2.1 were collected setting pinhole diameter at 4–5 Airy units, exciting the probe at 458 nm (80% Ar laser) and recording the emitted fluorescence at 470–500 nm and 530–570 nm respectively. Transmitted-light images were acquired simultaneously to confirm cell identity. Images were scanned at a resolution of 512×512 pixels and collected every 5 sec over a period of 15 to 30 minutes following treatment. Average YFP and CFP fluorescence intensities were calculated for each nucleus using Image J software. Values were then exported to a Microsoft Excel spread-sheet in order to calculate the YFP/CFP ratio for each time frame. Ratio values were then plotted over time to obtain a graphical representation of FRET intensity, corresponding to relative Ca^2+^ level variations in the nucleoplasm. Cells were counted as responsive when at least 2 peaks were present over 20 to 30 min imaging, the first peak always appearing within the first 15 min following the treatment. In case no peak was observed within the first 15 min, the cell was counted as not responsive and imaging was interrupted (fractions 60A and 60B). Thirty min imaging was systematically performed for fraction 2A tested on WT and the various mutant ROCs.

## Supporting Information

Figure S1 **^15^N-cross-polarization/magic angle spinning NMR analysis of** ***Aphanomyces euteiches*** **cell wall hydrolysate obtained after β-glucanase treatment.** The major signal at 121.2 ppm corresponds to the NH moiety of *N*-acetylglucosamine.
(PPTX)

Figure S2 **Expression of defense-associated genes in WT or mutant** ***Medicago truncatula*** **in response to glucan-chitosaccharide fractions.** Gene expression in the root system was analyzed by qRT-PCR after 4 h treatment of WT, *nfp-1* or *nfp-2* mutant seedlings with 100 µg.ml^−1^ fraction 60A, 60B, 2A, or 2B. Defense-associated gene expression was standardized in each sample using three reference genes encoding an histone H3, a translation elongation factor 1-α and a ubiquitin family protein/phosphatidylinositol 3,4-kinase, as described in [39]. Mean values from three biological replicates (± S.E.) are given as log2 of fold-expression in elicited seedlings with respect to mock-treated seedlings. The gene set used is the same as in Fig. 5. Asterisks above the bars indicate that the mutant line responded differently compared to the WT line (*P*<0.05). Such differences were observed only for the LOX and VR markers and did not concern both mutant lines, thereby showing that *NFP* is not involved in gene regulation in response to the glucan-chitosaccharide fractions.
(PPTX)

Table S1 **Glucanase activities present in culture filtrates of** ***Aphanomyces euteiches.*** Data are the mean±S.E. of 2 independent experiments. Filtrates of early stationary phase cultures were harvested 6 days post-inoculation. Glucanase activities were measured by incubation of culture filtrate aliquots with appropriate carbohydrate substrates and quantification of the liberated reducing ends as described in Materials and Methods.
(DOC)

## References

[1] Latgé JP (2010) Tasting the fungal cell wall. Cell Microbiol 12: 863–872. 20482553 10.1111/j.1462-5822.2010.01474.x

[2] Boller T, Felix G (2009) A renaissance of elicitors: perception of microbe-associated molecular patterns and danger signals by pattern-recognition receptors. Annu Rev Plant Biol 60: 379–406. 19400727 10.1146/annurev.arplant.57.032905.105346

[3] Tyler BM (2007) *Phytophthora sojae*: root rot pathogen of soybean and model oomycete. Mol Plant Pathol 8: 1–8. 20507474 10.1111/j.1364-3703.2006.00373.x

[4] Phillips AJ, Anderson VL, Robertson EJ, Secombes CJ, van West P (2008) New insights into animal pathogenic oomycetes. Trends Microbiol 16: 13–19. 18096392 10.1016/j.tim.2007.10.013

[5] Beakes GW, Glockling SL, Sekimoto S (2012) The evolutionary phylogeny of the oomycete “fungi”. Protoplasma 249: 3–19. 21424613 10.1007/s00709-011-0269-2

[6] Frey R (1950) Chitin und Zellulose in Pilzzellwänden. Ber Schweiz Bot Ges 60: 199–230.

[7] Bartnicki-García S (1968) Cell wall chemistry, morphogenesis and taxonomy of fungi. Annu Rev Microbiol 22: 87–108. 4879523 10.1146/annurev.mi.22.100168.000511

[8] Blum M, Boehler M, Randall E, Young V, Csukai M, et al (2010) Mandipropamid targets the cellulose synthase-like PiCesA3 to inhibit cell wall biosynthesis in the oomycete plant pathogen, *Phytophthora infestans* . Mol Plant Pathol 11: 227–243. 20447272 10.1111/j.1364-3703.2009.00604.x PMC6640402

[9] Blum M, Gamper HA, Waldner M, Sierotzki H, Gisi U (2012) The cellulose synthase 3 (CesA3) gene of oomycetes: structure, phylogeny and influence on sensitivity to carboxylic acid amide (CAA) fungicides. Fungal Biol 116: 529–542. 22483051 10.1016/j.funbio.2012.02.003

[10] Blum M, Gisi U (2012) Insights into the molecular mechanism of tolerance to carboxylic acid amide (CAA) fungicides in *Pythium aphanidermatum* . Pest Manag Sci 68: 1171–1183. 22431165 10.1002/ps.3279

[11] Lin CC, Aronson JM (1970) Chitin and cellulose in the cell walls of the oomycete, *Apodachlya sp* . Arch Mikrobiol 72: 111–114. 5469570 10.1007/BF00409517

[12] Bulone V, Chanzy H, Gay L, Girard V, Fèvre M (1992) Characterization of chitin and chitin synthase from the cellulosic cell wall fungus *Saprolegnia monoica* . Exp Mycol 16: 8–21.

[13] Campos-Takaki GM, Dietrich SMC, Mascarenhas Y (1982) Isolation and characterization of chitin from the cell walls of *Achlya radiosa* . J Gen Microbiol 128: 207–209.

[14] Badreddine I, Lafitte C, Heux L, Skandalis N, Spanou Z, et al (2008) Cell wall chitosaccharides are essential components and exposed patterns of the phytopathogenic oomycete *Aphanomyces euteiches* . Eukaryot Cell 7: 1980–1993. 18806214 10.1128/EC.00091-08 PMC2583540

[15] Werner S, Steiner U, Becher R, Kortekamp A, Zyprian E, et al (2002) Chitin synthesis during in planta growth and asexual propagation of the cellulosic oomycete and obligate biotrophic grapevine pathogen *Plasmopara viticola* . FEMS Microbiol Lett 208: 169–173. 11959432 10.1111/j.1574-6968.2002.tb11077.x

[16] Guerriero G, Avino M, Zhou Q, Fugelstad J, Clergeot P-H, et al (2010) Chitin synthases from *Saprolegnia* are involved in tip growth and represent a potential target for anti-oomycete drugs. PLoS Pathog 6: e1001070. 20865175 10.1371/journal.ppat.1001070 PMC2928807

[17] Grenville-Briggs L, Gachon CMM, Strittmatter M, Sterck L, Kupper FC, et al (2011) A molecular insight into algal-oomycete warfare: cDNA analysis of *Ectocarpus siliculosus* infected with the basal oomycete *Eurychasma dicksonii* . Plos One 6: 14. 10.1371/journal.pone.0024500 PMC3174193 21935414

[18] Gaulin E, Jacquet C, Bottin A, Dumas B (2007) Root rot disease of legumes caused by *Aphanomyces euteiches* . Mol Plant Pathol 8: 539–548. 20507520 10.1111/j.1364-3703.2007.00413.x

[19] Mélida H, Sandoval-Sierra JV, Diéguez-Uribeondo J, Bulone V (2013) Analyses of extracellular carbohydrates in oomycetes unveil the existence of three different cell wall types. Eukaryot Cell 12: 194–203. 23204192 10.1128/EC.00288-12 PMC3571302

[20] Cabib E, Durán A (2005) Synthase III-dependent chitin is bound to different acceptors depending on location on the cell wall of budding yeast. J Biol Chem 280: 9170–9179. 15637060 10.1074/jbc.M414005200

[21] Fontaine T, Sinenel C, Dubreucq G, Adam O, Delepierre M, et al (2000) Molecular organization of the alkali-insoluble fraction of *Aspergillus fumigatus* cell wall. J Biol Chem 275: 41528–41529. 11134062

[22] Kollár R, Petrakova E, Ashwell G, Robbins PW, Cabib E (1995) Architecture of the yeast cell wall. The linkage between chitin and beta(1→3)-glucan. J Biol Chem 270: 1170–1178. 7836376 10.1074/jbc.270.3.1170

[23] Silipo A, Erbs G, Shinya T, Dow JM, Parrilli M, et al (2010) Glyco-conjugates as elicitors or suppressors of plant innate immunity. Glycobiology 20: 406–419. 20018942 10.1093/glycob/cwp201

[24] Shibuya N, Minami E (2001) Oligosaccharide signalling for defence responses in plant. Physiol Mol Plant Pathol 59: 223–233.

[25] Oldroyd GE (2013) Speak, friend, and enter: signalling systems that promote beneficial symbiotic associations in plants. Nat Rev Microbiol 11: 252–263. 23493145 10.1038/nrmicro2990

[26] Kombrink A, Sánchez-Vallet A, Thomma BP (2011) The role of chitin detection in plant-pathogen interactions. Microbes Infect 13: 1168–1176. 21856436 10.1016/j.micinf.2011.07.010

[27] Gust AA, Willmann R, Desaki Y, Grabherr HM, Nürnberger T (2012) Plant LysM proteins: modules mediating symbiosis and immunity. Trends Plant Sci 17: 495–502. 22578284 10.1016/j.tplants.2012.04.003

[28] Gough C, Cullimore J (2011) Lipo-chitooligosaccharide signaling in endosymbiotic plant-microbe interactions. Mol Plant Microbe Interact 24: 867–878. 21469937 10.1094/MPMI-01-11-0019

[29] Maillet F, Poinsot V, André O, Puech-Pagès V, Haouy A, et al (2011) Fungal lipochitooligosaccharide symbiotic signals in arbuscular mycorrhiza. Nature 469: 58–63. 21209659 10.1038/nature09622

[30] Genre A, Chabaud M, Balzergue C, Puech-Pagès V, Novero M, et al (2013) Short-chain chitin oligomers from arbuscular mycorrhizal fungi trigger nuclear Ca^2+^ spiking in *Medicago truncatula* roots and their production is enhanced by strigolactone. New Phytol 198: 190–202. 23384011 10.1111/nph.12146

[31] Arrighi JF, Barre A, Ben Amor B, Bersoult A, Soriano LC, et al (2006) The *Medicago truncatula* lysin motif-receptor-like kinase gene family includes NFP and new nodule-expressed genes. Plant Physiol 142: 265–279. 16844829 10.1104/pp.106.084657 PMC1557615

[32] Fliegmann J, Uhlenbroich S, Shinya T, Martinez Y, Lefèbvre B, et al (2011) Biochemical and phylogenetic analysis of CEBiP-like LysM domain-containing extracellular proteins in higher plants. Plant Physiol Biochem 49: 709–720. 21527207 10.1016/j.plaphy.2011.04.004

[33] Nyamsuren O, Colditz F, Rosendahl S, Tamasloukht M, Bekel T, et al (2003) Transcriptional profiling of *Medicago truncatula* roots after infection with *Aphanomyces euteiches* (*Oomycota*) identifies novel genes upregulated during this pathogenic interaction. Physiol Mol Plant Pathol 63: 17–26.

[34] Djébali N, Jauneau A, Ameline-Torregrosa C, Chardon F, Jaulneau V, et al (2009) Partial resistance of *Medicago truncatula* to *Aphanomyces euteiches* is associated with protection of the root stele and is controlled by a major QTL rich in proteasome-related genes. Mol Plant Microbe Interact 22: 1043–1055. 19656040 10.1094/MPMI-22-9-1043

[35] Rey T, Nars A, Bonhomme M, Bottin A, Huguet S, et al (2013) NFP, a LysM protein controlling Nod factor perception, also intervenes in *Medicago truncatula* resistance to pathogens. New Phytol 198: 875–886. 23432463 10.1111/nph.12198

[36] van Loon LC, Rep M, Pieterse CMJ (2006) Significance of inducible defense-related proteins in infected plants. Annu Rev Phytopathol 44: 135–162. 16602946 10.1146/annurev.phyto.44.070505.143425

[37] Gaulin E, Madoui M-A, Bottin A, Jacquet C, Mathé C, et al (2008) Transcriptome of *Aphanomyces euteiches*: new oomycete putative pathogenicity factors and metabolic pathways. Plos One 3: e1723. 18320043 10.1371/journal.pone.0001723 PMC2248709

[38] Henrissat B, Davies G (1997) Structural and sequence-based classification of glycoside hydrolases. Curr Opin Struct Biol 7: 637–644. 9345621 10.1016/s0959-440x(97)80072-3

[39] Nars A, Rey T, Lafitte C, Vergnes S, Amatya S, et al (2013) An experimental system to study responses of *Medicago truncatula* roots to chitin oligomers of high degree of polymerization and other microbial elicitors. Plant Cell Rep 32: 489–502. 23314495 10.1007/s00299-012-1380-3

[40] Felix G, Duran JD, Volko S, Boller T (1999) Plants have a sensitive perception system for the most conserved domain of bacterial flagellin. Plant J 18: 265–276. 10377992 10.1046/j.1365-313x.1999.00265.x

[41] Vadassery J, Ranf S, Drzewiecki C, Mithöfer A, Mazars C, et al (2009) A cell wall extract from the endophytic fungus *Piriformospora indica* promotes growth of *Arabidopsis* seedlings and induces intracellular calcium elevation in roots. Plant J 59: 193–206. 19392691 10.1111/j.1365-313X.2009.03867.x

[42] Chabaud M, Genre A, Sieberer BJ, Faccio A, Fournier J, et al (2011) Arbuscular mycorrhizal hyphopodia and germinated spore exudates trigger Ca^2+^ spiking in the legume and nonlegume root epidermis. New Phytol 189: 347–355. 20880223 10.1111/j.1469-8137.2010.03464.x

[43] Burton RA, Fincher GB (2009) (1,3;1,4)-β-D-glucans in cell walls of the *Poaceae*, lower plants, and fungi: a tale of two linkages. Mol Plant 2: 873–882. 19825664 10.1093/mp/ssp063

[44] Chérif M, Benhamou N, Bélanger RR (1992) Occurrence of cellulose and chitin in the hyphal walls of *Pythium ultimum*: a comparative study with other plant pathogenic fungi. Can J Microbiol 39: 213–222.

[45] Asiegbu FO, Lonneborg A, Johansson M (1996) Chitin and glucans detected in the cell walls of *Pythium dimorphum*, an oomycetous fungus. Eur J Forest Pathol 26: 315–321.

[46] Bertke CC, Aronson JM (1992) Hyphal wall chemistry of *Lagenidium callinectes* and *Lagenidium chthamalophilum* . Botanica Marina 35: 147–152.

[47] Bertke CC, Aronson JM (1992) Hyphal wall composition of *Lagenidium giganteum.* . Mycologia 84: 571–574.

[48] Dietrich SM (1975) Comparative study of hyphal wall components of Oomycetes: *Saprolegniaceae* and *Pythiaceae* . An Acad Brasil Ciênc 47: 155–162.

[49] Yim NH, Il Hwang E, Yun BS, Park KD, Moon JS, et al (2008) Sesquiterpene furan compound CJ-01, a novel chitin synthase 2 inhibitor from *Chloranthus japonicus* SIEB. Biol Pharm Bull 31: 1041–1044. 18451544 10.1248/bpb.31.1041

[50] Ruiz-Herrera J, Gonzalez-Prieto JM, Ruiz-Medrano R (2002) Evolution and phylogenetic relationships of chitin synthases from yeasts and fungi. FEMS Yeast Res 1: 247–256. 12702327 10.1111/j.1567-1364.2002.tb00042.x

[51] Blank CE (2011) An expansion of age constraints for microbial clades that lack a conventional fossil record using phylogenomic dating. J Mol Evol 73: 188–208. 22105429 10.1007/s00239-011-9467-y

[52] Aziz A, Gauthier A, Bézier A, Poinssot B, Joubert JM, et al (2007) Elicitor and resistance-inducing activities of beta-1,4 cellodextrins in grapevine, comparison with beta-1,3 glucans and alpha-1,4 oligogalacturonides. J Exp Bot 58: 1463–1472. 17322548 10.1093/jxb/erm008

[53] Zhang B, Ramonell K, Somerville S, Stacey G (2002) Characterization of early, chitin-induced gene expression in *Arabidopsis* . Mol Plant Microbe Interact 15: 963–970. 12236603 10.1094/MPMI.2002.15.9.963

[54] Mithöfer A, Fliegmann J, Daxberger A, Ebel C, Neuhaus-Url G, et al (2001) Induction of H_2_O_2_ synthesis by beta-glucan elicitors in soybean is independent of cytosolic calcium transients. Febs Lett 508: 191–195. 11718714 10.1016/s0014-5793(01)03054-x

[55] Okinaka Y, Mimori K, Takeo K, Kitamura S, Takeuchi Y, et al (1995) A structural model for the mechanisms of elicitor release from fungal cell walls by plant beta-1,3-endoglucanase. Plant Physiol 109: 839–845. 8552716 10.1104/pp.109.3.839 PMC161384

[56] Cheong JJ, Birberg W, Fugedi P, Pilotti A, Garegg PJ, et al (1991) Structure-activity relationships of oligo-beta-glucoside elicitors of phytoalexin accumulation in soybean. Plant Cell 3: 127–136. 1840904 10.1105/tpc.3.2.127 PMC159985

[57] Fliegmann J, Mithöfer A, Wanner G, Ebel J (2004) An ancient enzyme domain hidden in the putative beta-glucan elicitor receptor of soybean may play an active part in the perception of pathogen-associated molecular patterns during broad host resistance. J Biol Chem 279: 1132–1140. 14578352 10.1074/jbc.M308552200

[58] Waldmüller T, Cosio EG, Grisebach H, Ebel J (1992) Release of highly elicitor-active glucans by germinating zoospores of *Phytophthora megasperma f. sp. glycinea* . Planta 188: 498–505. 24178381 10.1007/BF00197041

[59] Sieberer BJ, Chabaud M, Timmers AC, Monin A, Fournier J, et al (2009) A nuclear-targeted cameleon demonstrates intranuclear Ca^2+^ spiking in *Medicago truncatula* root hairs in response to rhizobial nodulation factors. Plant Physiol 151: 1197–1206. 19700563 10.1104/pp.109.142851 PMC2773104

[60] Zhu XH, Caplan J, Mamillapalli P, Czymmek K, Dinesh-Kumar SP (2010) Function of endoplasmic reticulum calcium ATPase in innate immunity-mediated programmed cell death. EMBO J 29: 1007–1018. 20075858 10.1038/emboj.2009.402 PMC2837167

[61] Lecourieux D, Ranjeva R, Pugin A (2006) Calcium in plant defence-signalling pathways. New Phytol 171: 249–269. 16866934 10.1111/j.1469-8137.2006.01777.x

[62] Ranf S, Eschen-Lippold L, Pecher P, Lee J, Scheel D (2011) Interplay between calcium signalling and early signalling elements during defence responses to microbe- or damage-associated molecular patterns. Plant J 68: 100–113. 21668535 10.1111/j.1365-313X.2011.04671.x

[63] Navazio L, Baldan B, Moscatiello R, Zuppini A, Woo SL, et al (2007) Calcium-mediated perception and defense responses activated in plant cells by metabolite mixtures secreted by the biocontrol fungus *Trichoderma atroviride* . BMC Plant Biol 7: 41. 17663762 10.1186/1471-2229-7-41 PMC1950503

[64] Francia D, Chiltz A, Lo Schiavo F, Pugin A, Bonfante P, et al (2011) AM fungal exudates activate MAP kinases in plant cells in dependence from cytosolic Ca^2+^ increase. Plant Physiol Biochem 49: 963–969. 21561784 10.1016/j.plaphy.2011.04.008

[65] Leclercq J, Fliegmann J, Tellström V, Niebel A, Cullimore JV, et al (2008) Identification of a multigene family encoding putative β-glucan-binding proteins in *Medicago truncatula* . J Plant Physiol 165: 766–776. 17728012 10.1016/j.jplph.2007.02.008

[66] Ben Amor B, Shaw SL, Oldroyd GED, Maillet F, Penmetsa RV, et al (2003) The *NFP* locus of *Medicago truncatula* controls an early step of Nod factor signal transduction upstream of a rapid calcium flux and root hair deformation. Plant J 34: 495–506. 12753588 10.1046/j.1365-313x.2003.01743.x

[67] Pauly N, Knight MR, Thuleau P, Graziana A, Muto S, et al (2001) The nucleus together with the cytosol generates patterns of specific cellular calcium signatures in tobacco suspension culture cells. Cell Calcium 30: 413–421. 11728136 10.1054/ceca.2001.0250

[68] Rupley JA (1964) The hydrolysis of chitin by concentrated hydrochloric acid, and the preparation of low-molecular-weight substrates for lysozyme. Biochim Biophys Acta 83: 245–255. 14236697 10.1016/0926-6526(64)90001-1

[69] Somogyi M (1952) Notes on sugar determination. J Biol Chem 195: 19–23. 14938350

[70] Lowry OH, Rosebrough NJ, Farr AL, Randall RJ (1951) Protein measurement with the Folin phenol reagent. J Biol Chem 193: 265–275. 14907713

[71] Selvendran RR, Stevens BJH, O’Neil MA (1985) Developments in the isolation and analysis of cell walls from edible plants. In: Seminar series Brett CT, Hillman JR, editors. Biochemistry of plant cell walls. Society for Experimental Biology. 28: 39–78.

[72] Ciucanu I, Kerek F (1984) A simple and rapid method for the permethylation of carbohydrates. Carbohydr Res 131: 209–217.

[73] Heux L, Brugnerotto J, Desbrières J, Versali MF, Rinaudo M (2000) Solid state NMR for determination of degree of acetylation of chitin and chitosan. Biomacromolecules 1: 746–751. 11710206 10.1021/bm000070y

[74] Miyawaki A, Llopis J, Heim R, McCaffery JM, Adams JA, et al (1997) Fluorescent indicators for Ca^2+^ based on green fluorescent proteins and calmodulin. Nature 388: 882–887. 9278050 10.1038/42264

