# Supplementary figures and images for: Aphanomyces euteiches Cell Wall Fractions Containing Novel Glucan-Chitosaccharides Induce Defense Genes and Nuclear Calcium Oscillations in the Plant Host Medicago truncatula
Source: PLoS One. 2013 Sep 23;8(9):e75039. doi: 10.1371/journal.pone.0075039 (PMC3781040; doi:10.1371/journal.pone.0075039)

## Slide 1
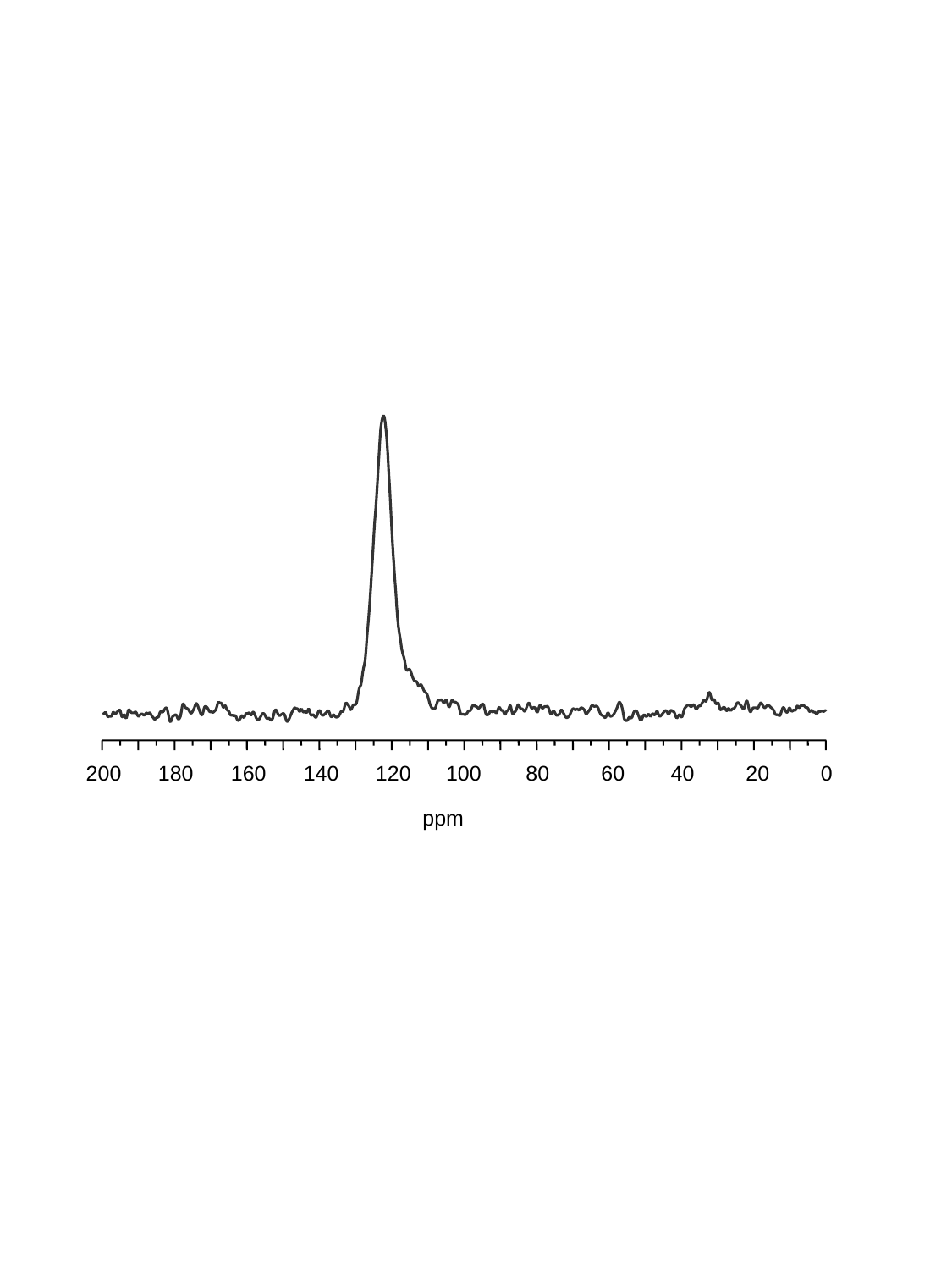

200
180
160
140
120
100
80
60
40
20
0
ppm

Supplement: Figure S1 — 15N-cross-polarization/magic angle spinning NMR analysis of Aphanomyces euteiches cell wall hydrolysate obtained after β-glucanase treatment. The major signal at 121.2 ppm corresponds to the NH moiety of N-acetylglucosamine. (PPTX) [file pone.0075039.s001.pptx]

## Slide 1
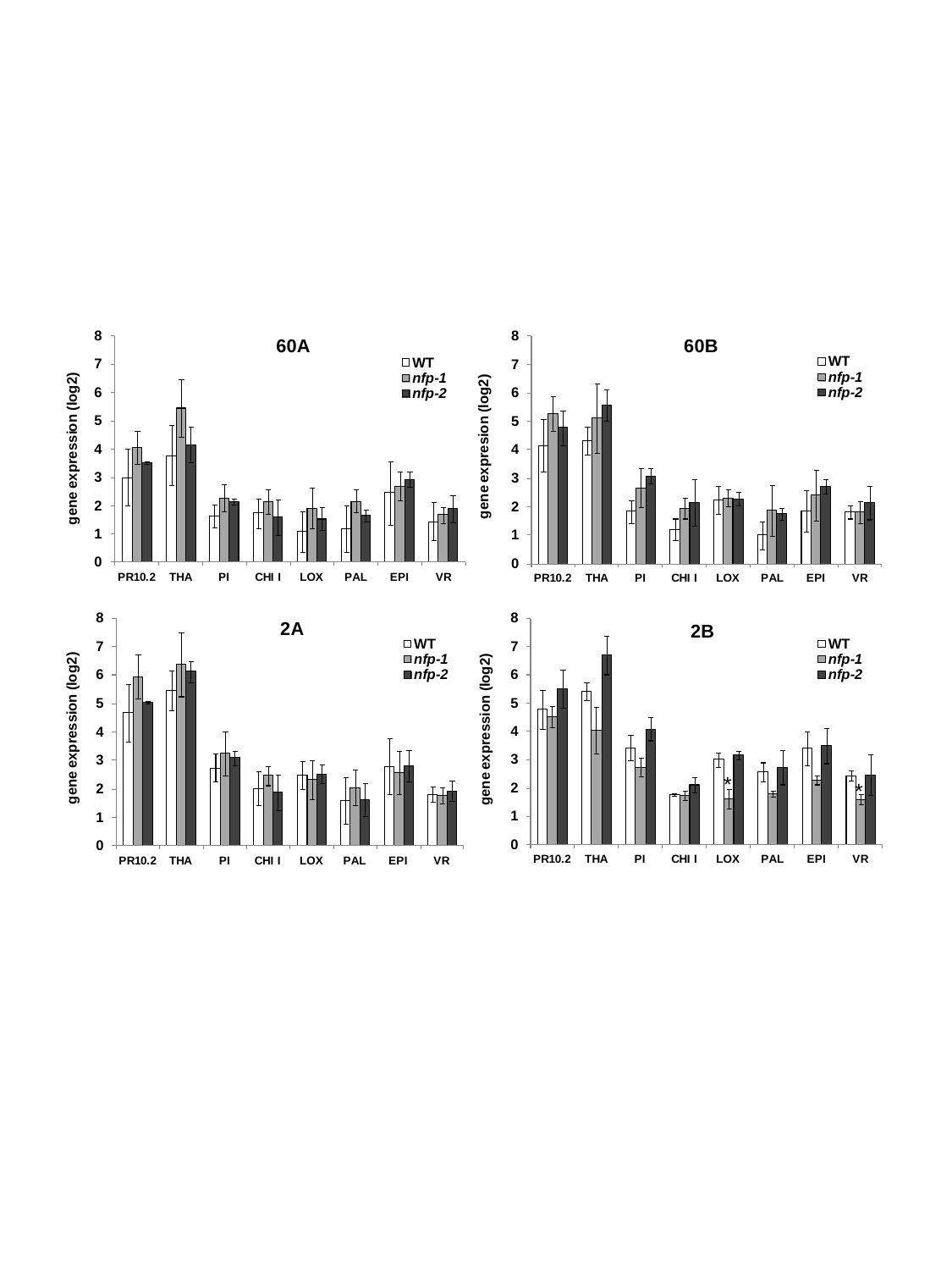

*
*

Supplement: Figure S2 — Expression of defense-associated genes in WT or mutant Medicago truncatula in response to glucan-chitosaccharide fractions. Gene expression in the root system was analyzed by qRT-PCR after 4 h treatment of WT, nfp-1 or nfp-2 mutant seedlings with 100 µg.ml−1 fraction 60A, 60B, 2A, or 2B. Defense-associated gene expression was standardized in each sample using three reference genes encoding an histone H3, a translation elongation factor 1-α and a ubiquitin family protein/phosphatidylinositol 3,4-kinase, as described in [39]. Mean values from three biological replicates (± S.E.) are given as log2 of fold-expression in elicited seedlings with respect to mock-treated seedlings. The gene set used is the same as in Fig. 5. Asterisks above the bars indicate that the mutant line responded differently compared to the WT line (P<0.05). Such differences were observed only for the LOX and VR markers and did not concern both mutant lines, thereby showing that NFP is not involved in gene regulation in response to the glucan-chitosaccharide fractions. (PPTX) [file pone.0075039.s002.pptx]
