# Supplementary material for: Aphanomyces euteiches Cell Wall Fractions Containing Novel Glucan-Chitosaccharides Induce Defense Genes and Nuclear Calcium Oscillations in the Plant Host Medicago truncatula
Source: PLoS One. 2013 Sep 23;8(9):e75039. doi: 10.1371/journal.pone.0075039 (PMC3781040; doi:10.1371/journal.pone.0075039)
Supplement: Table S1 — Glucanase activities present in culture filtrates of Aphanomyces euteiches. Data are the mean±S.E. of 2 independent experiments. Filtrates of early stationary phase cultures were harvested 6 days post-inoculation. Glucanase activities were measured by incubation of culture filtrate aliquots with appropriate carbohydrate substrates and quantification of the liberated reducing ends as described in Materials and Methods. (DOC) [file pone.0075039.s003.doc]

**Table S1. Glucanase activities present in culture filtrates of *Aphanomyces euteiches*^a^.**

|  | Specific activity (nkat.mg^-1^) |
| --- | --- |
| β-1,4-glucanase | 1.83 + 0.18 |
| β-1,3-glucanase | 8.70 + 2.30 |
| β-1,6-glucanase | 0.83 + 0.03 |

^a^ Data are the mean + S.E. of 2 independent experiments. Filtrates of early stationary phase cultures were harvested 6 days post-inoculation. Glucanase activities were measured by incubation of culture filtrate aliquots with appropriate carbohydrate substrates and quantification of the liberated reducing ends as described in Materials and Methods.
